# MoFap7, a ribosome assembly factor, is required for fungal development and plant colonization of *Magnaporthe oryzae*

**DOI:** 10.1080/21505594.2019.1697123

**Published:** 2019-12-09

**Authors:** Lin Li, Xue-Ming Zhu, Huan-Bin Shi, Xiao-Xiao Feng, Xiao-Hong Liu, Fu-Cheng Lin

**Affiliations:** a State Key Laboratory for Rice Biology, Biotechnology Institute, Zhejiang University, Hangzhou, China; b State Key Laboratory for Rice Biology, China National Rice Research Institute, Hangzhou, China

**Keywords:** MoFap7, ribosome assembly factor, fungal development, virulence, Pmk1 pathway

## Abstract

Fap7, an important ribosome assembly factor, plays a vital role in pre-40S small ribosomal subunit synthesis in *Saccharomyces cerevisiae* via its ATPase activity. Currently, the biological functions of its homologs in filamentous fungi remain elusive. Here, MoFap7, a homologous protein of ScFap7, was identified in the rice blast fungus *Magnaporthe oryzae*, which is a devastating fungal pathogen in rice and threatens food security worldwide. Δ*Mofap7* mutants exhibited defects in growth and development, conidial morphology, appressorium formation and infection, and were sensitive to oxidative stress. In addition, site-directed mutagenesis analysis confirmed that the conserved Walker A motif and Walker B motif in MoFap7 are essential for the biological functions of *M. oryzae*. We further analyzed the regulation mechanism of MoFap7 in pathogenicity. MoFap7 was found to interact with MoMst50, a regulator functioning in the MAPK Pmk1 signaling pathway, that participates in modulating plant penetration and cell-to-cell invasion by regulating the phosphorylation of MoPmk1. Moreover, MoFap7 interacted with the GTPases MoCdc42 and MoRac1 to control growth and conidiogenesis. Taken together, the results of this study provide novel insights into MoFap7-mediated orchestration of the development and pathogenesis of filamentous fungi.

## Introduction

*Magnaporthe oryzae* is a filamentous plant pathogenic fungus that infects many kinds of crops including rice and barley, and causes rice blast and wheat blast. It is a model organism for studying the interaction between plants and pathogens [1,2]. Appressoria, specialized infection structures, are produced by *M. oryzae* to penetrate host plant cells and cause infection [3]. The mitogen-activated protein kinase (MAPK) cascade is evolutionarily conserved and extremely important for more than 20 pathogenic fungi [4–6]. However, the pathogenic mechanism of MAPK is different in plants and animals [7].The MAPK cascade responds to host and environmental signals in *M. oryzae*. Three MAPK pathways are involved in regulating invasive growth, appressprium formation, cell wall integrity, and surface recognition in *M. oryzae* [8]. MoMps1 is essential for plant infection, and the Δ*Momps1* mutant is defective in appressoria penetration. The MoMps1 is activated by upstream MoMck1 and MoMkk2. MoMst50 acts as an adaptor of MoMck1-MoMkk2 interaction, but MoMkk2 interacts with MoMps1 through its MAPK docking site [4,8,9]. MoMig1 and MoSwi6 are the main downstream of MoMps1. The Δ*Momig1* mutant is defective in the differentiation and growth of invasive hyphae. The Δ*Moswi6* mutants have defects in cell wall integrity, mycelial growth, and appressorium penetration [8,10,11]. Furthermore, the cyclic adenosine monophosphate-protein kinase A (cAMP-PKA) pathway regulates various developmental and infective processes. MoMsb2, MoSho1, MoPth11 and MoCbp1 are involved in the recognition of extracellular or surface signals to activate downstream cAMP-PKA pathways [12–14].

In *M. oryzae*, MoPmk1 is essential for regulating appressorium formation and invasive growth. A Δ*Mopmk1* mutant of *M. oryzae* is unable to form appressoria, and cause diseases [15]. MoPmk1 phosphorylation and kinase activity are required for appressorium development on both artificial and plant surfaces. Recent studies have found that inhibition of MoPmk1 inhibits the ability of *M. oryzae* to spread from a plant cell to adjacent plant cells, allowing fungal infection to occur only in a single plant cell [16]. In *M. oryzae*, the PMK1 pathway includes MoMst11, MoMst7, MoMst50, MoMst12 and MoSfl1 [17, 18; 19]. In the Pmk1 signaling cascade, MoMst7 and MoMst11 function upstream of MoPmk1 [19]. Two transcription factors, MoMst12 and MoSfl1, act downstream of the PMK1 signaling pathway and are essential for the invasive growth of *M. oryzae* [17,20]. Δ*Momst11* and Δ*Momst50* mutants have consistent defects in appressorium formation, are sensitive to osmotic stresses, and are nonpathogenic [18]. In *M. oryzae*, MoMst50 is an important component of the PMK1 signaling pathway, and Δ*Momst50* mutants have similar defects to Δ*Mopmk1* mutants in terms of appressorium formation and plant infection, functioning as adaptor proteins in the MoMst11-MoMst7-MoPmk1 cascade [18,19]. The sterile alpha-motif (SAM) domain of MoMst50, rather than the Ras-association domain (RAD), is indispensable for appressorium formation and for the interaction of MoMst50 with MoMst11 [18]. Additionally, MoMst50 interacts with MoCdc42 and MoMgb1 [18], suggesting that MoMst50 may be involved and play a key role in signaling pathways other than the Pmk1 pathway.

The term small GTPases refers to members of the protein superfamily of small guanosine triphosphatases, also known as small G protein or the Ras superfamily, which are involved in almost every aspect of cell biology [21]. Small GTPases function via a binary on/off status by controlling the loading (activation) of GTP and the hydrolysis of GTP to GDP (inactivation). In eukaryotes, the superfamily includes five conserved families: Ras, Rho, Rab, Arf, and Ran [21]. Rho GTPases primarily regulate actin cytoskeletal dynamics, cell shape and cell polarity. Previous studies have determined the importance of strict spatial regulation of Rho, Rac and Cdc42 functions by these family members through combined activities of GEFs and though controlled cytoskeletal activity and subsequent morphogenesis [22,23,24]. In *S. cerevisiae*, Cdc42 functions in the transduction of polarity signals to the morphogenetic machinery [25,26]. In pathogenic fungi such as *Claviceps purpurea, Ustilago maydis* and *Colletotrichum trifolii*, deletion of Cdc42 results in abnormal morphology of certain cell types, such as conidia, and nonpathogenicity to the plant host [27,28]. In *M. oryzae*, MoCdc42 plays a key role in growth, infection, conidial morphology and pathogenicity. Recent studies have found that MoCdc42 is also involved in appressorium-mediated infection processes in *M. oryzae* [29]. Rac1 is also a member of the Rho family of GTPases. In rice, the Rac homolog OsRac1 plays a role in disease resistance by activating reactive oxygen intermediate (ROI) production and cell death [30]. In the pathogenic fungus *C. trifolii*, Rac1 regulates MAPK activation and production of intracellular reactive oxygen species (ROS) and restores the mycelial morphology of dominant Ras mutants [31]. In the pathogenic fungus *U. maydis*, Rac1 is essential for pathogenicity as well as conidial morphology and mycelial growth [27]. In *M. oryzae*, Δ*Morac1* deletion mutants are defective in conidial production, and the MoRac1-MoChm1 pathway is responsible for conidiogenesis [32]. Chm1 is a typical structural component of p21-activated kinases (PAKs), with a conserved catalytic domain and a p21-Rho-binding domain (PBD) or Cdc42-Rac interactive binding (CRIB) domain. In *S. cerevisiae*, PAKs are involved in various cellular signaling and developmental processes as effectors of Rho-family GTPases, including Rho, Rac, and Cdc42 [33,34].

Cellular proteins are synthesized in ribosomes. Eukaryotic ribosomes are complex ribonucleoprotein particles usually composed of ~80 ribosomal proteins and four RNA molecules. In addition, more than 200 different auxiliary ribosomal assembly factor proteins and ~70 small nucleolar RNAs (snoRNAs) are involved in ribosome assembly [35–37]. Many of these auxiliary factors belong to different classes of GTPases and ATPases [38,39]. One example of such a factor is a protein called Fap7 in *S. cerevisiae* [40] and adenylate kinase 6 (hADK6) in humans [41]. In *S. cerevisiae*, Fap7 was first screened by activating Pos9-dependent reporter genes under oxidative stress [40]. Fap7 is important for the assembly of small ribosomal subunits with unusual dual ATPase and adenylate kinase activities [42, 43].

Since Fap7 is an important protein with dual functions of adenylate kinase and GTPase activity, the null mutant in yeast is lethal [44]. For the first time, we obtained null mutants of MoFap7 in *M. oryzae* using the target-gene replacement method. We found that MoFap7 is conserved and involved in responding to oxidative stress and interacting with the ribosomal protein MoRps14, as found previously for its homolog Fap7 in yeast. Here, we reported that MoFap7 is involved in regulating growth, conidial morphology, appressorium formation, penetration and pathogenicity in this fungal pathogen. MoFap7 interacts with MoCdc42 and MoRac1, modulating growth and conidiogenesis. Importantly, MoFap7 is able to interact with MoMst50, regulating the phosphorylation of MoPmk1, which is important for plant infection and cell-to-cell invasion [16].

## Results

### Identification of MoFap7

**Figure 1.**
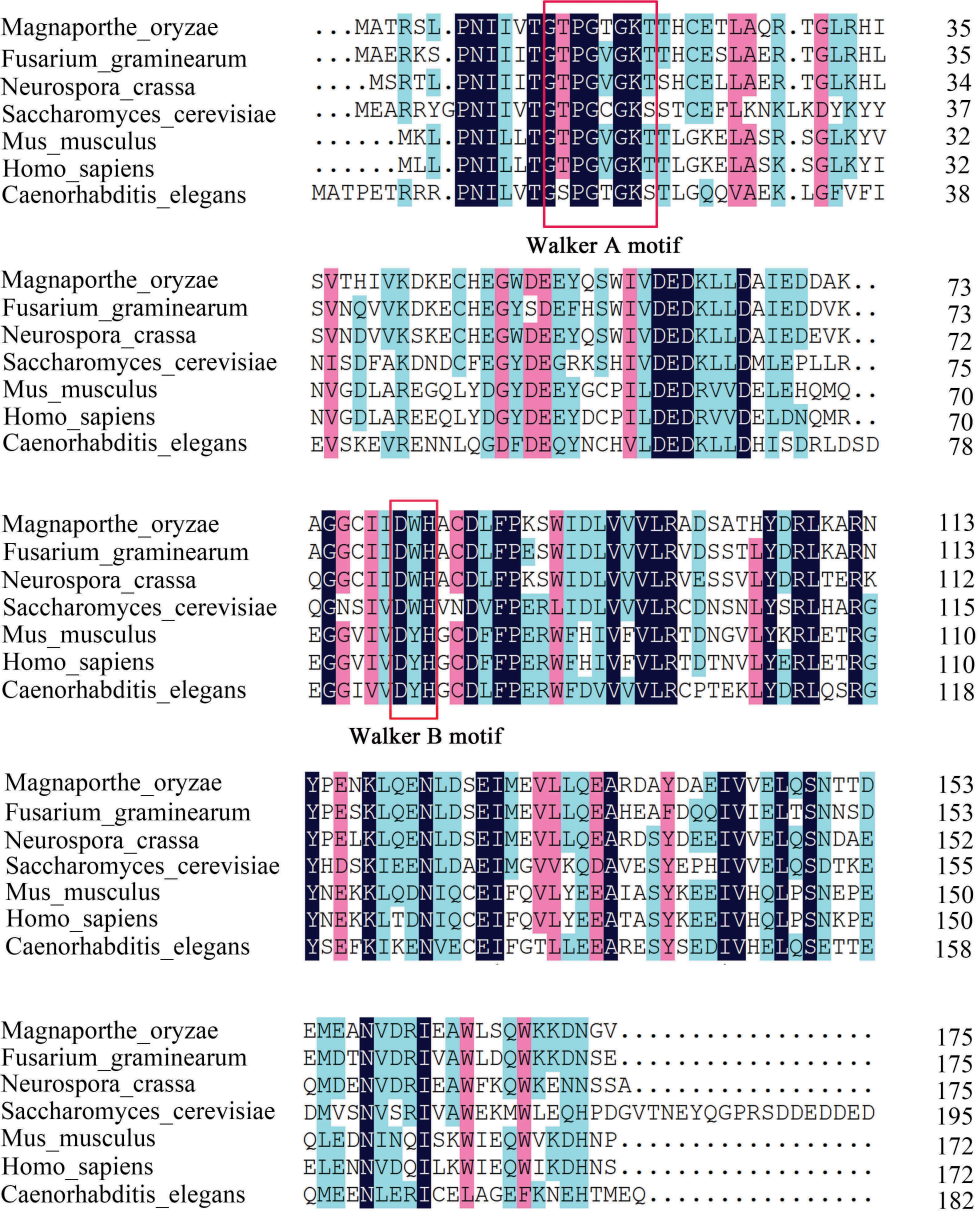
Comparison of amino acids of Fap7 and homologues in different eukaryotes. Using CLUSTALW to align the amino acids of Fap7 in *Magnaporthe oryzae* (MoFap7), *Fusarium graminearum* (XP_011327243.1), *Neurospora crassa* (XP_001728530.2), *Saccharomyces cerevisiae* (NP_010115.1), *Mus musculus* (NP_081868.1), *Caenorhabditis elegans* (NP_496065.1) and *Homo sapiens* (NP_057367.1). All of these Fap7 proteins have a conserved Walker A motif and a Walker B motif (red framed part).

To explore roles of MoFap7 in the development of *M. oryzae, MoFAP7* (MGG_16212) was identified by performing BLASTP searches in the *M. oryzae* genome database (http://fungi.ensembl.org/Magnaporthe_oryzae/Tools/Blast?db=core) based on the protein sequences of the yeast counterpart Fap7. Sequence alignment revealed that the MoFap7 amino acid sequence has a high degree of sequence similarity to Fap7 homologs in other organisms (Figure 1). MoFap7 shows high similarity (51%) to Fap7 in yeast, 48.1% similarity to its homolog in mammals, and 46.2% similarity to its homolog in *C. elegans*. Yeast Fap7 contains the Walker A and B sequence elements of an ATPase but structurally resembles an adenylate kinase (AK) [45,46]. MoFap7 also harbors a GhhhhGK[T/S] Walker A motif as well as an hhh(DE)XH-type Walker B motif (h represents any hydrophobic residue). The sequence alignment indicated that Fap7 is conserved from fungi to mammals.

### MoFap7 interacts with MoRps14 and is mainly localized in the nucleus

**Figure 2.**
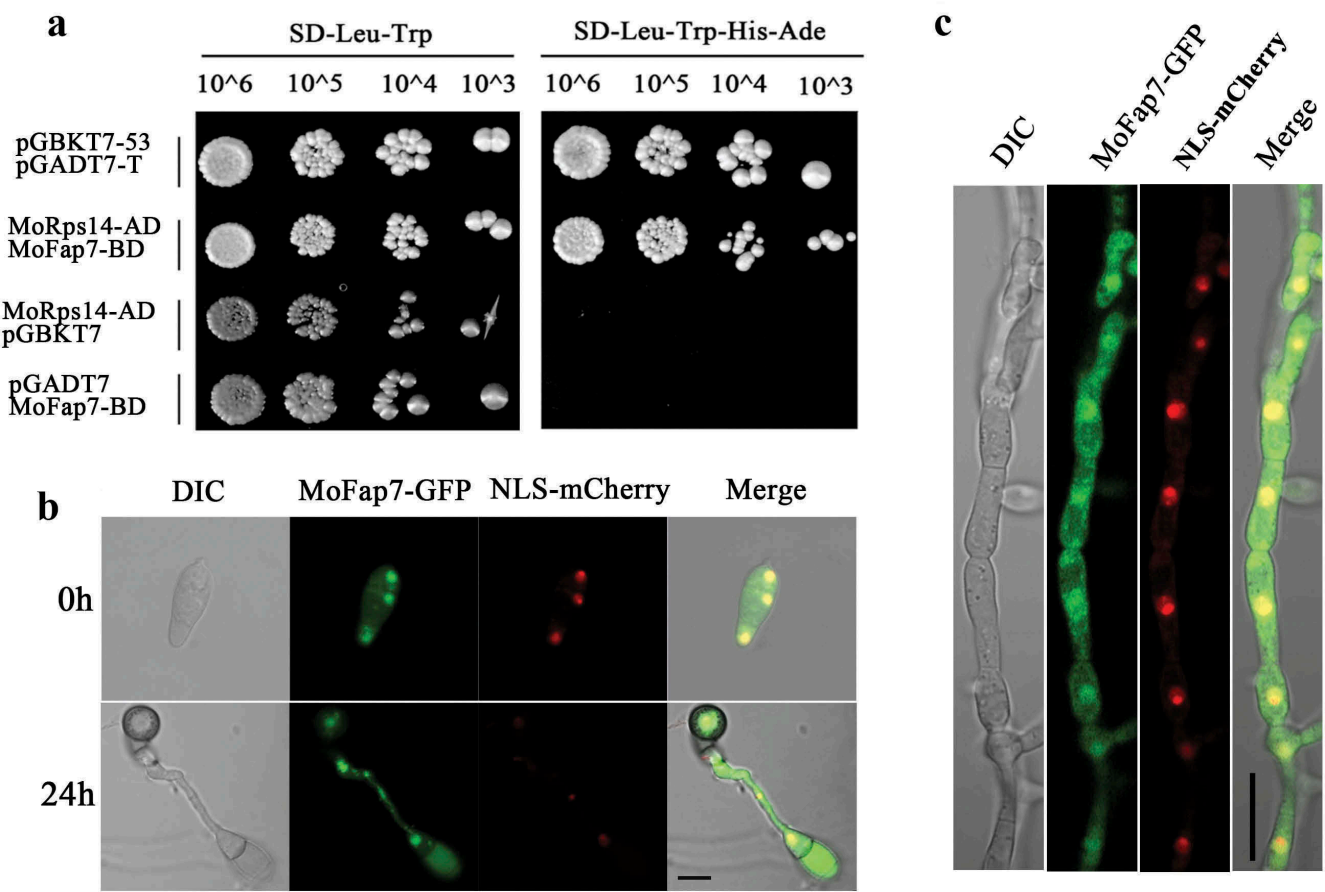
MoFap7 interacts with MoRps14, and dynamic subcellular localization of MoFap7. (a) Yeast two-hybrid analysis. The pair of plasmids pGBKT7-53 and pGADT7-T was used as the positive control. The pairs of plasmids pGBKT7- and MoRps14-AD, pGADT7 and MoFap7-BD were used as the negative control. (b) MoFap7-GFP conidia expressing NLS-mCherry was inoculated on plastic cover slips and incubated in a moist chamber before microscopic observations. (c) DIC and epifluorescence images were captured at the indicated time points. MoFap7-GFP conidia expressing NLS-mCherry were grown in liquid CM medium at 25°C for 24 h. Scale bar, 10 μm.

In yeast, Fap7 specifically interacts with the ribosomal protein Rps14 to participate in the biosynthesis of the small subunit. To determine whether MoFap7 also participates in the same process, interaction between MoFap7 and MoRps14 were detected by a yeast two-hybrid assay. Similarly, we also found that MoFap7 interacts with MoRps14 in *M. oryzae* (Figure 2(a)) indicating that MoFap7 has conserved functions in small subunit biosynthesis. However, the binding domain of MoFap7 does not interact with activation domain with MoFap7. MoRps14 is the same (Fig. S1).

To characterize the intracellular localization of MoFap7, a vector containing MoFap7-GFP was constructed and transformed into the Δ*Mofap7* mutant. When observed under a fluorescence microscope, MoFap7-GFP appeared in the nucleus. To investigate the spatial and temporal distribution of MoFap7 during the appressoria formation process, an NLS-mCherry (a nuclear location signal) fusion protein was co-expressed with MoFap7-GFP. Conidia were harvested from 8-day-old colonies grown on CM plates and incubated in H_2_O on the surface of a hydrophobic film to allow germ tube and appressorium development. Most notably, each cell of conidium or appressorium contained a single fluorescent puncta overlapping with the NLS-mCherry signal(Figure 2(b)). We found that MoFap7 is mainly localized in the nucleus in hyphae(Figure 2(c)). These results confirmed that MoFap7 is predominantly localized to the nucleus in *M. oryzae*.

### MoFap7 is important for growth, conidial morphology and conidiation

**Figure 3.**
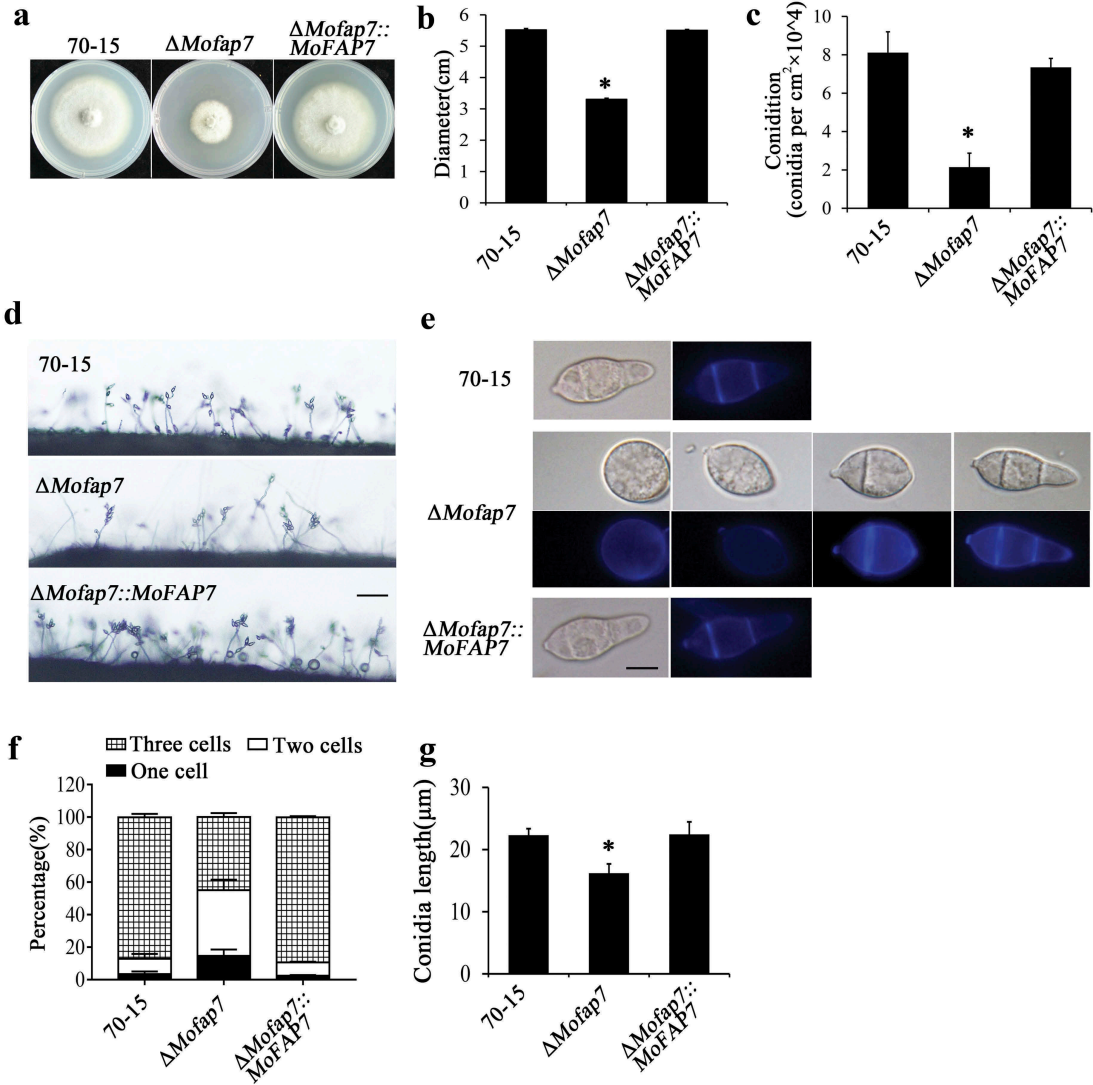
MoFap7 is important for growth and conidiation. (a) 70–15, Δ*Mofap7* mutant and the MoFap7 complemented strain were grown on CM medium for 8 days.(b) Statistical analysis of the diameters of hyphae from the wild-type 70–15, the Δ*Mofap7* mutant, and the complemented strains on CM.(c) Statistical analysis of conidia production.(d) Images of conidia and conidiophores observed under a light microscope after induction of conidiation in the dark at 28°C. Samples were incubated for conidia or conidiophores developed in the Δ*Mofap7* mutant and the complemented strains. Scale bar, 100 μm.(e) Conidial septa of 70–15, Δ*Mofap7* mutant and the MoFap7 complemented strain stained with CFW. Bars, 10 μm.(f) Numbers of conidial cells in of 70–15, Δ*Mofap7* mutant and the MoFap7 complemented strain.(g) Statistical analysis of length of 70–15, Δ*Mofap7* mutant and the MoFap7 complemented strain.Asterisks denote statistical significances (P < 0.01).

To characterize the functions of MoFap7, a Δ*Mofap7* deletion mutant was generated by replacing the coding region with a glufosinate ammonium resistance cassette (BAR) (Fig. S2). Compared with the wild-type strain 70–15 and the complemented strain (Δ*Mofap7::MoFAP7*), the Δ*Mofap7* mutant showed significantly attenuated growth and a reduction in mycelial growth of approximately 45% (Figure 3(a,b)).

We then evaluated the functions of Δ*Mofap7* in conidiophores and conidiation. Quantitative analysis revealed that conidiation of the mutant strains was dramatically reduced (<30% of that in WT and complemented strain) (Figure 3(d,c)). Consistent with this result, we observed that the number of conidia per conidiophore was reduced in Δ*Mofap7* mutants (Figure 3(c,d)). Comparison of the morphology of conidia produced by the tested strains indicated that the length of Δ*Mofap7* mutant conidia was 16.20 ± 1.04 μm, significantly shorter than that of conidia from the WT and complemented strains (22.32 ± 1.04 μm and 22.45 ± 2.01 μm respectively) (Figure 3(g)). The length-to-width ratio of the mutant conidia was 2.07 (and was 2.84 and 2.85 for the WT and complemented conidia, respectively); thus, Δ*Mofap7* mutant conidia were more globose than control conidia. More interestingly, over 50% of conidia formed by these mutants had one septum or no septa, in contrast with conidia formed by the wild-type strain (Figure 3(e)). The conidial morphology of the complemented strain was restored to normal (Figure 3(f)). Taken together, these results suggested that MoFap7 is required for vegetative growth and conidiogenesis in *M. oryzae*.

### MoFap7 affects appressorium formation and virulence

**Figure 4.**
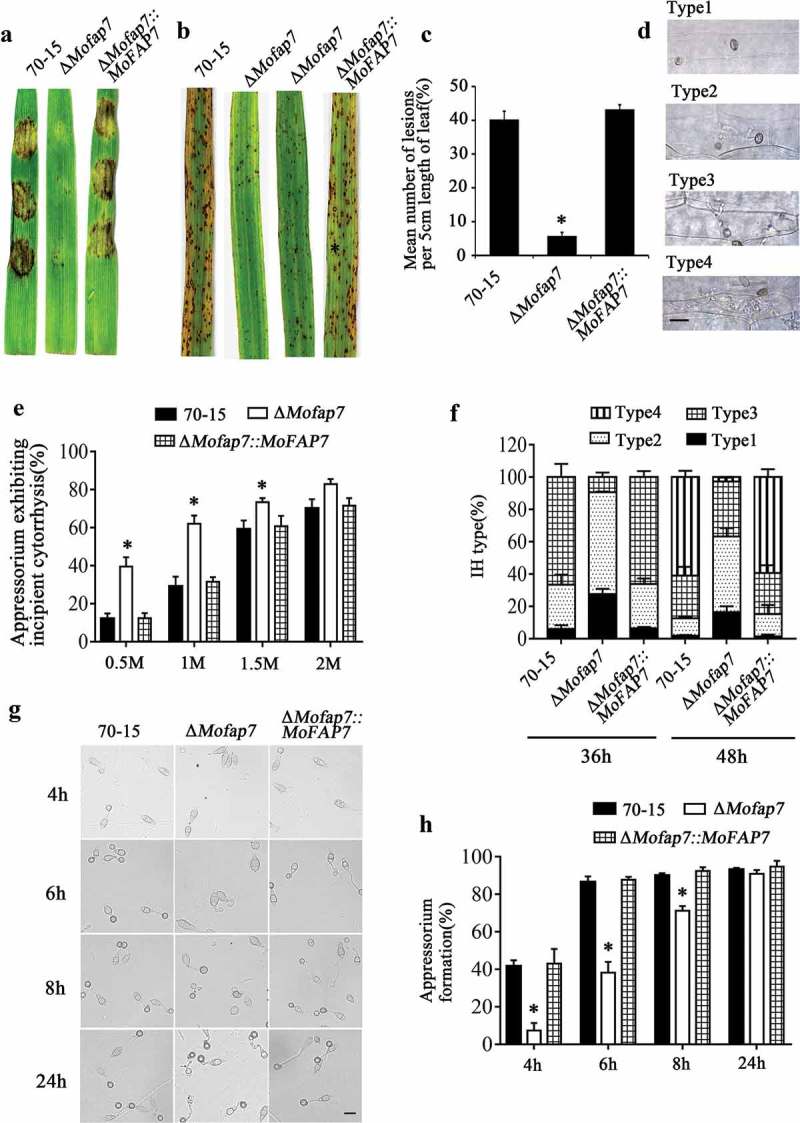
MoFap7 is important for appressoria formation and virulence. (a) Disease symptoms on cut leaves of barley inoculated with mycelial plugs from 70–15, the Δ*Mofap7* mutant and the complemented strain. Typical leaves were photographed 4 days after inoculation.(b) Two-week-old rice seedlings were inoculated by spraying with 5 × 10^4^ conidia/ml conidia suspensions from 70–15, the Δ*Mofap7* mutant and the complemented strain. Lesion formation on rice leaves was evaluated 7 days after inoculation.(c) Quantification of the lesion numbers per 5 cm length of rice leaf. Error bars represent SD and asterisk represents significant difference (P < 0.01).(d) and (f) The infectious hyphae were divided into four types. For each strain tested, 100 hyphae were counted per replicate and the experiment was repeated three times. Scale bar, 10 μm.(e) Different concentrations of glycerol were used for cytorrhysis analysis. For each concentration, at least 100 appressoria were counted and the experiment was repeated three times with the same result. Asterisks denote statistical significances (P < 0.01).(g) Appressorium formation assay on the hydrophobic surfaces. Scale bar, 10 μm.(h) Appressorium formation rates were calculated and statistically analyzed. Asterisks represent significant differences (P < 0.01).

On the hydrophobic surface, the Δ*Mofap7* mutant showed delayed appressorium development compared with that of wild-type 70–15 and the complemented strains (Figure 4(g,h)) while this delay became indistinguishable after 24 h. Considering that appressoria of Δ*Mofap7* form slowly but tend to be normal after 10 hours, we speculated that the slowed of infection is associated with turgor pressure. Incipient cytorrhysis assays [11] were performed using a glycerol concentration of 0.5–2 M. As shown in Figure 4(e), the collapse rate of Δ*Mofap7* mutant appressoria was significantly higher than that of appressoria of the wild-type 70–15 or the complemented strains. The results indicated that MoFap7 affects the appressorium formation and turgor pressure.

To further examine the functions of MoFap7 in pathogenesis, we performed assays on two hosts (barley and rice). Mycelial plugs of the 70–15, Δ*Mofap7*, and complemented strains were inoculated on detached barley leaves. Mycelial plugs of the 70–15 and complemented strains caused severe lesions (Figure 4(a)). However, mycelial plugs of the Δ*Mofap7* mutant produced small, restricted lesions. Similarly, conidial suspensions (5 × 10^4^ conidia/ml) were sprayed onto susceptible rice seedlings (*Oryza sativa* cv. CO-39). Only a few small brown spots (not typical blast lesions) were observed on leaves infected with the Δ*Mofap7* mutant (Figure 4(b)). The lesions produced by Δ*Mofap7* were also smaller and less than those produced by the 70–15 and complemented strains (Figure 4(c)).

To gain further insight into the virulence reduction in Δ*Mofap7*, we performed penetration assays using detached barley leaves. Invasive hyphae (IH) for each strain at 36 h post-inoculation (hpi) and 48 hpi were classified into 4 types (type 1, no hyphal penetration; type 2, IH with one or two branches; type 3, short and modestly extended IH with at least three branches; type 4, IH that have numerous branches and fully occupy a plant cell) (Figure 4(d)). The four types of IH from the various strains were quantified and statistically analyzed. We found that in the 70–15 and complemented strains, nearly 70% of IH were type 3; in contrast, less than 10% of IH were type 3 in Δ*Mofap7* at 36 hpi. At 48 hpi, only 2.62% of the IH in Δ*Mofap7* were type 4, while more than 50% of IH in the wild-type and complemented strains were type 4 (Figure 4(f)). Infection experiments showed that compared to wild type, the mutant exhibited arrested growth in individual cells and extended slowly to neighboring cells. These results suggested that MoFap7 is important for appressorium mediated infection, invasive hyphal growth and virulence.

### The walker A and walker B motifs of MoFap7 are indispensible for its biological functions

**Figure 5.**
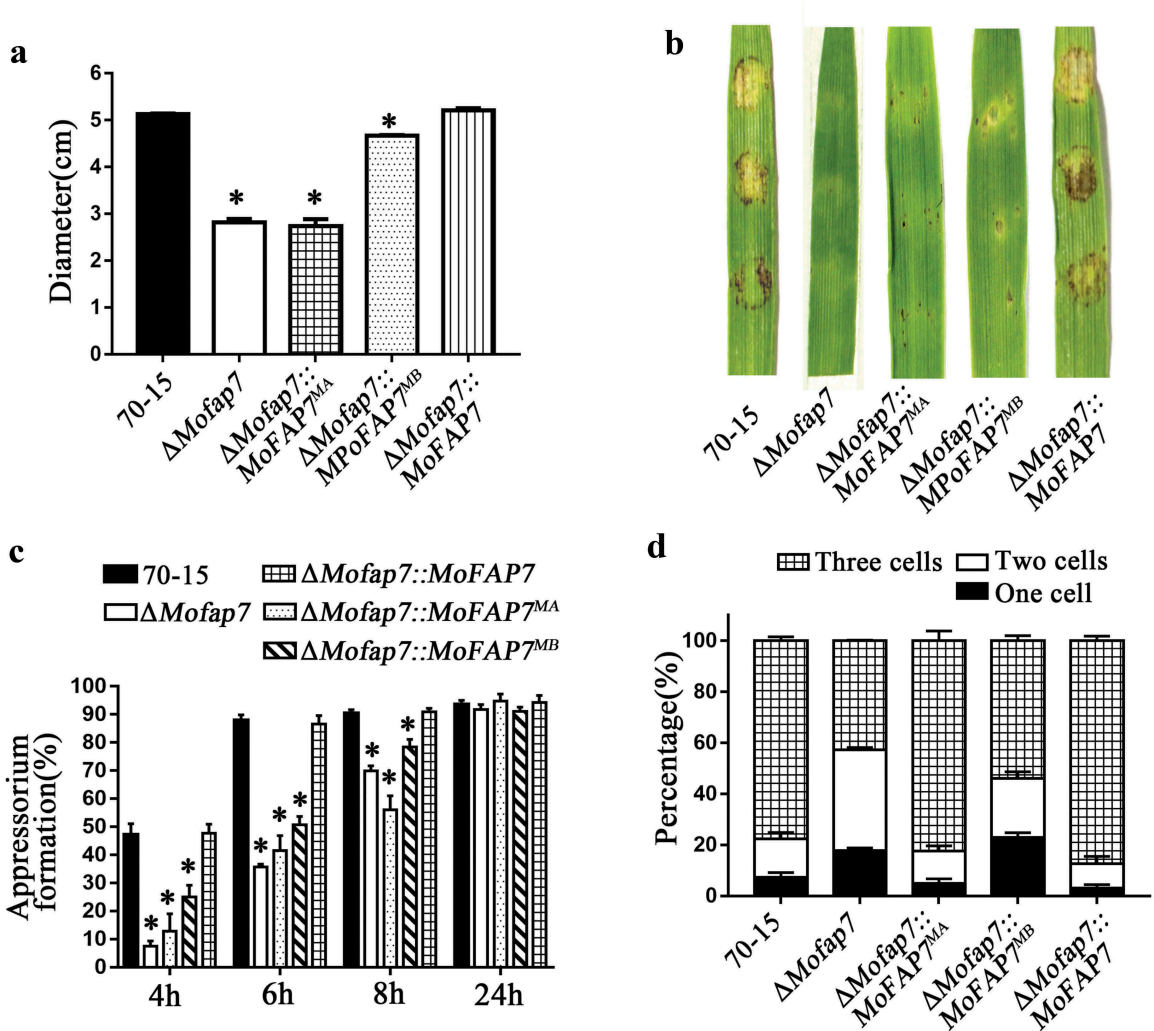
Phenotype of Δ*Mofap7::MoFAP7^MA^* and Δ*Mofap7::MoFAP7^MB^* strains. (a) 70–15, ΔM*ofap7::MoFAP7^MA^*, Δ*Mofap7::MoFAP7^MB^* and the MoFap7 complemented strain were grown on CM medium.(b) Symptoms of 4 days barley leaves inoculated with 70–15, Δ*Mofap7::MoFAP7^MA^*, Δ*Mofap7::MoFAP7^MB^* and Δ*Mofap7::MoFAP7* strains.(c) The rate of appressorium formation was calculated at 4 hours, 6 hours, 8 hours, and 24 hours of induction and statistical analysis was performed.(d) Numbers of conidial cells in of 70–15, Δ*Mofap7::MoFAP7*, Δ*Mofap7::MoFAP7^MA^* and Δ*Mofap7::MoFAP7^MB^* strains.

Fap7 is predicted to belong to a family of P-loop-type kinases, which harbor putative Walker A, which is predicted to bind the α-phosphate of ribonucleoside substrates [47] and hhh(DE)XH-type Walker B motifs characteristic of NTPases [40,47]. The Walker A motif binds the β- and γ-phosphate moieties of nucleoside triphosphates (NTPs), whereas the Walker B motif contacts the essential Mg^2+^ cofactor. As shown in Figure 1, the amino acid sequence of MoFap7 is highly conserved from fungi to mammals. We analyzed whether the highly conserved amino acids in the motif of MoFap7 are essential for its biological function in *M. oryzae*. Six highly conserved residues G13/P15/G16/G18/K19/T21 in the Walker A motif were mutated into alanine together as a mutated allele *MoFAP7^MA^* or individually. In addition, another two highly conserved residues, D82/H84, in the Walker B motif were also mutated to alanine together forming mutated allele *MoFAP7^MB^*. The site mutation strains were verified by genome sequencing. Sequence alignments indicated that the target sites have been changed (Fig. S3). To explore the insertion site of the site-directed mutagenesis, we performed high-efficiency thermal asymmetric interlaced PCR (hiTAIL PCR) as described in Table S4. As shown in Fig. S8, every site mutation sequence was inserted in the non-coding region of the *M. oryzae* genome. In addition, we verified the copy number of the site mutant strains by qPCR, and the sequences were all single-copy insertion in the genome (Fig. S8). These results confirmed that the defects of the site mutation strains are caused by the site-directed mutagenesis in the motifs. Further growth phenotypes of transformants containing these point mutations were determined. Fig. S4 shows that the Δ*Mofap7::MoFAP7^MB^* strain was approximately about 91% that of the wild-type strain in *M. oryzae*. In comparison with the wild type, the growth rate of the Δ*Mofap7::MoFAP7^MA^* strain was only 53% (Figure 5(a)), while strains carrying the MoFap7 G18A and K19A mutations also showed a significantly reduced growth rates (Fig. S4).

Conidial morphology was determined by observing the conidia collected from 9-day-old cultures on CM plates. Almost 90% of the conidia in the complemented strains and Δ*Mofap7::MoFAP7^MA^* strains were normal, but only 50% of the Δ*Mofap7::MoFAP7^MB^* conidia were normal with two septa (Figure 5(d)). We then examined conidial germination and found that the appressoria of Δ*Mofap7::MoFAP7^MA^* and Δ*Mofap7::MoFAP7^MB^* formed slowly in the early stage but did not differ significantly from those of the complemented strains after 8 hours (Figure 5(c)). As shown in Figure 5(b), the virulence of Δ*Mofap7::MoFAP7^MA^* and Δ*Mofap7::MoFAP7^MB^* was reduced compared to that of the complemented strain. These conclusions are consistent with those in yeast [40,43], indicating that the Walker A and Walker B motifs of MoFap7 are conserved and important for the biological functions of MoFap7.

### Deletion of MoFap7 increases sensitivity to oxidative stress

**Figure 6.**
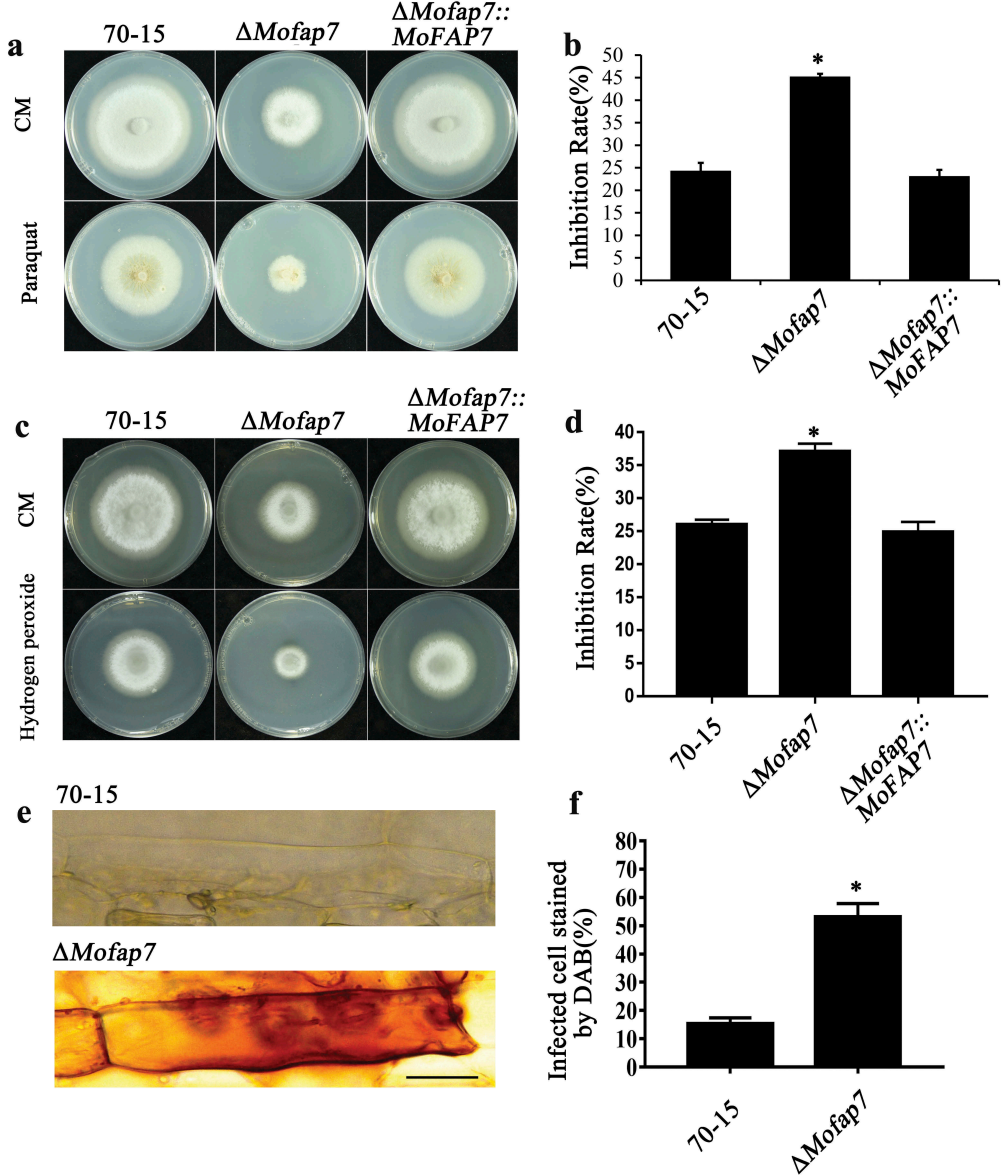
MoFap7 is involved in oxidative stress. (a) and (c) Sensitivity test of oxidative stress Paraquat and Hydrogen peroxide. Strains were incubated on CM supplemented with 5 mM Paraquat or 1 mM Hydrogen peroxide at 28°C for 8 days. (b) and (d) The inhibition rate was determined by plotting the percentage of colonies in the presence of various stresses against the control. Asterisks denote statistical significances (P < 0.01). (e) 3, 3ʹ-Diaminobenzidine (DAB) staining assays of the penetrated plant cells. Barley epidermis was stained with DAB at 32 hpi and observed. Bars, 50 μm. (f) The infected cell stained by DAB. Three independent biological experiments were performed, with three replicates each time and yielded similar results in each independent biological experiment. Error bars represent standard deviation, and asterisks represent significant difference between the different strains (p < 0.01).

The *fap7-1* mutant in yeast is sensitive to hydrogen peroxide [40]. To evaluate the roles of MoFap7 in mediating *M. oryzae* adaptation to pathogenesis-associated stresses, we compared the radial growth rates of the WT, Δ*Mofap7* and complemented strains on CM containing the oxidative stress agents hydrogen peroxide (H_2_O_2_) and paraquat. As shown in Figure 6(a,b), the Δ*Mofap7* mutant exhibited increased sensitivity to paraquat. In addition, the Δ*Mofap7* mutant exhibited increased sensitivity to hydrogen peroxide(H_2_O_2_) (Figure 6(c,d)). Our findings suggested that disruption of MoFap7 results in increased sensitivity to oxidative stress.

The accumulation of reactive oxygen species (ROS) in host cells when infected by wild type and deletion mutants was also examined by staining with 3, 3ʹ-diaminobenzidine (DAB) at 32 hpi. Barley cells infected with WT hyphae were not stained with DAB, whereas barley cells infected by Δ*Mofap7* mutant hyphae were strongly stained with DAB (Figure 6(e,f)). Taken together, these results indicated that MoMap7 plays roles in scavenging host-derived ROS.

### MoFap7 affects the phosphorylation of MoPmk1 by interacting with MoMst50

**Figure 7.**
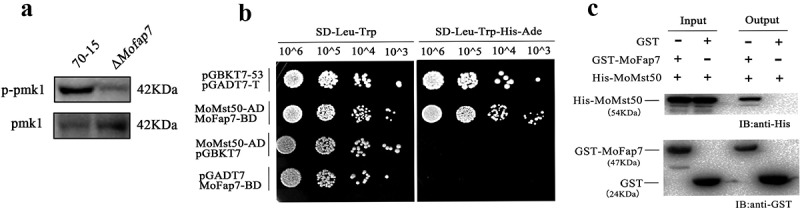
MoFap7 affects the phosphorylation of MoPmk1 by interacting with MoMst50. (a) Total proteins were isolated from mycelia of 70–15 and Δ*Mofap7* mutant. Pmk1phosphorylation level was detected by applying phosphor-Pmk1 antibody. Endogenous Pmk1 level was detected by the Pmk1 antibody. (b) Yeast two-hybrid assay for examining the interaction between MoFap7 and MoMst50. The transformants expressing AD-MoMst50 and empty BD, empty AD and BD-MoFap7 were used as negative control. (c) Protein binding assay for MoFap7-MoMst50 interaction in vitro. GST-beads were used to bind GST protein (24 kDa) or GST-tagged MoFap7 protein (47 kDa), respectively, and incubated with His-tagged MoMst50 protein (54 kDa). Total eluted fractions from the beads (output) were immunoblotted with the His and GST antibodies.

In *M. oryzae*, MoPmk1 is reported to be extremely important for appressorium formation and invasive growth [8]. The latest research further confirmed that MoPmk1 could regulate rice blast fungus expansion into adjacent plant cells [16]. The Δ*Mofap7* mutant showed a similar cell-to-cell invasion phenotype. Therefore, we examined the effect of MoFap7 on MoPmk1 phosphorylation. As shown in Figure 7(a), the phosphorylation level MoPmk1 was significantly reduced in the Δ*Mofap7* mutant compared to that in wild-type 70–15.

To further explore the mechanism by which MoFap7 regulates the phosphorylation level of MoPmk1, we screened MoFap7-interacting proteins by yeast two-hybrid screening system. A construct pGBKT7-Fap7 and a *M. oryzae* cDNA library were co-introduced into AH109 yeast cells. Transformed cells were cultured on SD/-Trp/-Leu/-His plates at 30°C for 3–5 days followed by selection of colonies and further incubation on SD/-Trp/-Leu/-His/-Ade X-α-gal for screening blue colonies. By sequencing and analyzing the blue colonies, MoMst50 was found to be a candidate protein that interacted with MoFap7 (Table S2). We then further validated the interaction between MoFap7 and MoMst50 using yeast two-hybrid analysis. Transformants expressing BD-MoFap7 and AD-MoMst50 constructs could grow on SD-Leu-Trp-His-Ade plates (Figure 7(b)), indicating that MoFap7 interacts with MoMst50. Furthermore, the interaction was verified by an in vitro GST pull-down assay. The recombinant protein GST-MoFap7 could pull down His-MoMst50 but GST could not (Figure 7(c)). The results of Pull down with Ladder in Fig. S5A. These results indicated that MoFap7 is involved in regulating the of phosphorylation level of MoPmk1, by interacting with MoMst50 which is an important scaffold of the Pmk1 signaling pathway.

### MoFap7 interacts with two small GTP-binding proteins

**Figure 8.**
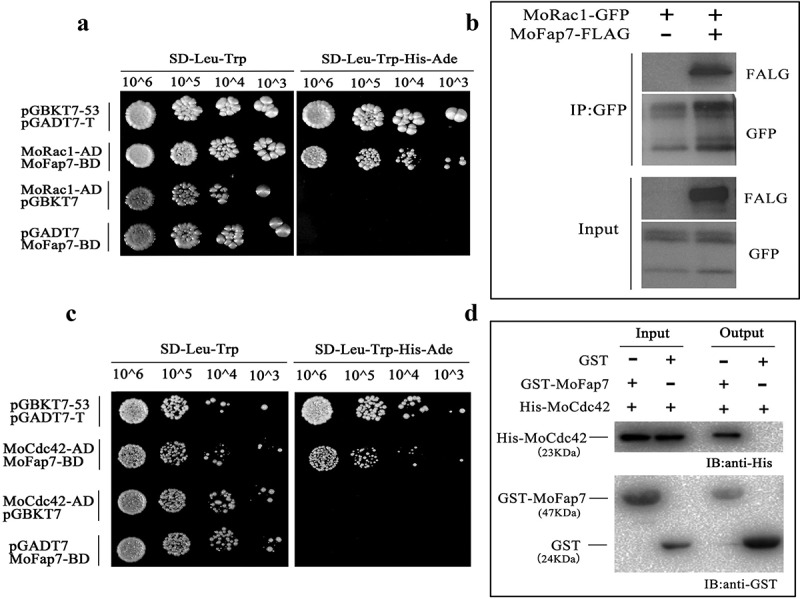
MoFap7 interacts with MoRac1 and MoCdc42. (a) and (c) Yeast two-hybrid analysis. The pair of plasmids pGBKT7–53 and pGADT7-T was used as the positive control. Plates were incubated at 30°C for 3 days before being photographed.(c) Co-immunoprecipitation assay. Western blots of total proteins (total) and proteins eluted from anti-GFP agarose (elution) of the transformants expressing the MoFap7 fusion with GFP. The total proteins (total) isolated from transformants co-expressing the MoFap7-GFP and MoRac1-3×FLAG. The presence of MoFap7 and MoRac1 was detected with an anti-GFP and an anti-FLAG antibody respectively.(d) In vitro pull down assays. The recombinant GST-MoFap7 or GST bound to glutathione Sepharose beads was incubated with *E. coli* cell lysate containing His-MoCdc42. Eluted proteins were analyzed by immunoblot (IB) with the monoclonal anti-His and anti-GST antibodies.

To explore the potential mechanism of MoMap7, we employed protein immunoprecipitation (IP) to identify putative MoFap7-interacting proteins. We generated a MoFap7-3× FLAG construct and introduced it into the Δ*Mofap7* mutant. After measuring MoFap7 expression, we immobilized MoFap7 to anti-FLAG M2 beads and analyzed proteins co-purified with FLAG tagged MoFap7 by mass spectrometry (MS). It is worth noting that we found some of the ribosome-associated proteins and NTPases in the MS data, including MoRps14, MoRho1, MoCdc15, MoSep3 and MoSep6 (Table S1). In the yeast two-hybrid screening library, the potential interacting proteins were MoRho1, MoRac1, and MoCdc42, all of which belong to the family of small G proteins (Table S2). Subsequently, we verified that MoFap7 interacts with MoRac1 and MoCdc42 by yeast two-hybrid analysis (Figure 8(a,b,c)). We demonstrated that MoFap7 interacts with MoRac1 through Co-IP experiments (Figure 8(c,b)), and demonstrates that MoFap7 interacts with MoRac1 and MoCdc42 through Pull-down experiments (Figure 8(d) and Fig. S5B). Fig. S5C shows the Pull-down results with Ladder. Our data showed that MoFap7 directly interacts with MoRac1 and MoCdc42. To explore the effect of site mutations on interactions. The interactions of Δ*Mofap7::MoFAP7^MA^*, Δ*Mofap7::MoFAP7^MB^* with MoRac1, MoCdc42, MoMst50 were verified by yeast two-hybrid assay. We found that MoRac1, MoCdc42 and Δ*Mofap7::MoFAP7^MA^*, Δ*Mofap7::MoFAP7^MB^* did not interact each other. MoMst50 does not interact with Δ*Mofap7::MoFAP7^MB^* and has a weak interaction with Δ*Mofap7::MoFAP7^MA^* (Fig. S6). The results indicated that the Walker A motif is no more important for interaction with MoMst50, MoRac1, and MoCdc42 than the Walker B motif, although the Δ*Mofap7::MoFAP7^MA^* mutant appears to have more serious defects in growth. It seems that both the Walker A motif and the Walker B motif have no effect on interaction.

## Discussion

Fap7 belongs to the adenylate kinase family, and its biological function is to catalyze the reversible transfer of a phosphate group from adenosine triphosphate (ATP) to adenosine monophosphate (AMP) to form two molecules of adenosine diphosphate (ADP) [48]. AKs play an important role in nucleotide metabolism. However, the biological function of this enzyme in filamentous fungi is still mysterious. From archaea to eukaryotes, the interaction of Fap7 with Rps14 is conserved and participates in the biosynthesis of small ribosomal subunits [43]. In our research, MoFap7 was shown to physically interact with MoRps14, as evidenced by the yeast two-hybrid assay results. Subsequently, we obtained null mutants of MoFap7 in *M. oryzae*, although deletion of its homolog in yeast was lethal. Although MoFap7 is a ribosome assembly factor, we discovered that MoFap7 affects the development and pathogenicity of *M. oryzae* for the first time. The subcellular localization of MoFap7 was mainly in the nucleus during conidial germination and appressoria formation. Δ*Mofap7* exhibited increased sensitivity to oxidative stress. In addition, the conserved Walker A and Walker B motifs in MoFap7 play important roles in growth, appressorium formation and pathogenicity.

In *S. cerevisiae*, Fap7 is an essential nuclear and cytoplasmic protein previously identified in a genetic screen for factors required for the activation of a reporter construct during oxidative stress [40, 49]. In the rice blast fungus, strains exhibited significantly slowed growth and were sensitive to oxidative stresses after *MoFAP7* knockout. This finding is consistent with the observation that the *fap7-1* mutant in yeast is sensitive to oxidative stress and exhibits slow growth on glucose [40]. The predominantly nuclear localization of MoFap7 in *M. oryzae* is also consistent with that in yeast. In *Caenorhabditis elegans*, knockdown of the cAK6 gene results in growth suspension [50]. In human cells, hCINAP (also known as AK6) is required for human 18S rRNA processing and 40S subunit assembly [45]. In yeast, compared to wild-type strains, strains carrying the Fap7 K20R (Walker A motif) and D82AH84A (Walker B motif) mutations showed significantly reduced growth rates and the involvement of conserved amino acids in nucleotide binding, and NTP enzyme activity is essential for in vivo Fap7 function [42]. In our study, lysine(K) at position 19 of MoFap7 corresponding to the lysine at position 20 of the yeast ScFap7 is conserved. The strains *M. oryzae* and yeast grow slow after mutating this site. The growth of the D82AH84A mutant strain was slightly slowed. Interestingly, the growth of *M. oryzae* was severely affected by mutation of the six conserved amino acids in the Walker A motif. These findings indicated that the Walker A motif and the Walker B motif of MoFap7 are conserved and extremely important in *M. oryzae*.

It has been reported that Fap7 has unusual dual ATPase and adenylate kinase activity [43]. Deregulation of the ADP/ATP ratio can interfere with Fap7-Rps14 binding and/or dissociation and affect ribosomal biogenesis [48]. Rps14 strongly activates the ATPase activity of Fap7, but does not activate its adenylate kinase activity, and Rps14 acts as a ribosomal protein to activate the ATPase activity [43]. Intriguingly, knockout of MoFap7 significantly reduced the phosphorylation level of MoPmk1.Therefore, MoFap7 may assist in the transfer of ATP to MoPmk1 through interaction with MoMst50. However, the nature of MoFap7 as an adenylate kinase in *M. oryzae* is not currently understood, and a more in-depth study of its underlying mechanisms is needed.

**Figure 9.**
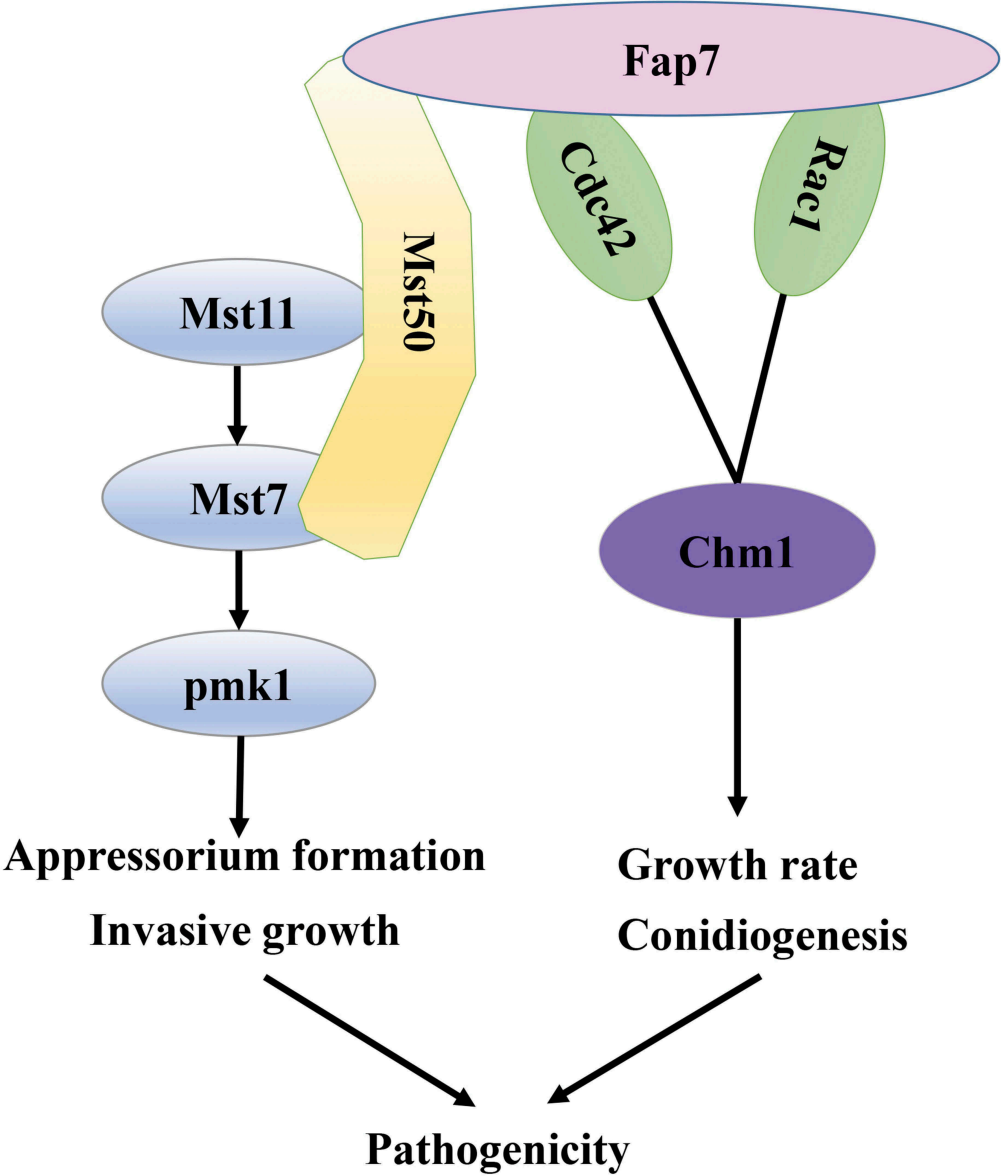
A proposed model of MoFap7 in the regulation of *M. oryzae* virulence. MoFap7 interacts with MoMst50 affect the phosphorylation level of MoPmk1. MoFap7 interacts with MoRac1 and MoCdc42 to regulate the growth and conidiogenesis and affect pathogenicity.

MoFap7 affects the phosphorylation level of MoPmk1 by interacting with MoMst50. According to previous studies, MoPmk1 affects *M. oryzae* appressorium formation and controls mycelial expansion to adjacent plant cells and pathogenicity [16]. MoMst50 is an adaptor protein in the MoMst11-MoMst7-MoPmk1 cascade, which is essential for appressorium formation [18]. The Δ*Mofap7* mutant is similar to the Δ*Mopmk1* mutant in terms of invasive growth. Both mutants are blocked in invasive growth although Fap7 is not necessary for the formation of appressoria. We demonstrated for the first time that MoFap7 affects the phosphorylation of MoPmk1 by interacting with MoMst50, affecting appressorium formation, invasive growth and pathogenicity. In addition, we found that MoFap7 interacts with the small G proteins MoRac1 and MoCdc42. It has been reported that MoMst50 interacts with MoCdc42 in *M. oryzae*, and that MoCdc42 is required for infection, conidial morphogenesis and virulence in *M. oryzae* [18,29]. In *M. oryzae*, MoMst50 interacts with MoChm1, although MoChm1 plays either no role or only a minor role in MoPmk1 activation [9]. The ortholog of MoCdc42 is similar to MoChm1, which is a putative component of the Pmk1 signaling pathway and is independent of appressoria formation but is important for the morphogenesis and function of *M. oryzae* [18,29,51]. The growth of the Δ*Morac1* mutant is slowed, the conidia are deformed, the formation of the appressoria is defective, and the pathogenicity is reduced [9,32]. Chm1 is an effector of Rac1 and Cdc42 [32]. In *M. oryzae*, MoChm1 is extremely important for growth, conidia morphology and pathogenicity. The MoRac1-MoChm1 pathway in *M. oryzae* is responsible for conidiogenesis [32]. We have reason to infer that MoFap7 controls pathogenicity via the Pmk1 pathway and GTP-binding proteins (Figure 9).

In summary, our research provides new insights into the functions of the ribosome assembly factor MoFap7 in fungal pathogenesis by regulating penetration and invasive growth. With MoMst50, MoFap7 regulates appressorium formation, invasive growth, oxidative stresses response, and pathogenicity. In synergy with MoRac1 and MoCdc42, MoFap7 regulates growth, conidiation, and conidial morphology. The site-directed mutagenesis analysis results indicate that the Walker A motif and the Walker B motif are extremely important for the function of MoFap7.

## Experimental procedures

### Fungal strains and culture conditions

*M. oryzae* 70–15 was used as the wild type strain in this study and all strains were cultured on complete medium (CM) agar plates at 25°C [52]. DNA or RNA was exacted from mycelia grown in liquid CM for two days.

### Targeted gene deletion and complementation

All deletion mutants were generated according to a high-throughput gene knockout method described previously [53,54]. In this study, 1.4-kb upstream/downstream flanking sequences of MoFap7 were amplified from *M. oryzae* genomic DNA with -specific primers (Supporting Information Table S3), The 0.9-kb glufosinate ammonium (BAR) sequence was amplified from pCB1003 [55] with primer pair BAR-F/BAR-R. Through a fusion enzyme, these three fragments [] were ligated with the HindIII/XbaI-linearized vector pKO1B [53]. The targeted gene-deletion vector was introduced into the wild-type strain 70–15 using *Agrobacterium tumefaciens*-mediated transformation (ATMT) [56]. The copy number of the selective marker gene BAR in the genome of the mutant was confirmed by quantitative PCR (qPCR), and the β-tubulin gene (one copy in the genome) was used as a control. If the copy number of BAR is calculated to be 1.0 ± 0.2, the mutant is a single inserted gene containing the selective marker gene [54]. Mutants confirmed by Southern blotting analysis (Fig. S7). To construct plasmids for generating complemented strains, the full-length genomic gene with the 2-kb native promoter sequence was amplified from *M. oryzae* genomic DNA and cloned into the linearized vector pKD5 [53] using a fusion enzyme (Vazyme, China). The resulting constructs were transformed into the gene-deletion mutants using the ATMT strategy.

### Phenotype assays

Development of conidiophores and conidia was monitored as reported by Lau and Hamer (1998)[57], and agar blocks with marginal mycelial of the colonies were cultured under successive illumination levels for 24 h. For conidia production, mycelia were cultured on CM agar plates at 25°C with a 16 h light and 8 h dark cycle for 9 days. Conidia were harvested from three mycelial plates by washing with 3 ml of sterile water and were counted with a hemocytometer under a microscope. Conidial germination and appressorium formation assays were conducted on plastic coverslips at 4, 6, 8 and 24 hpi. To examine appressorium-mediated penetration and invasive growth, 20 μl of a 5 × 10^4^ conidia/ml conidial suspension was inoculated on the surface of barley for 36h and 48h, after which the surface was decolorized before microscopic observation according to the previously described protocol [54]. To assess the pathogenicity of the strains on rice, 3 ml of a conidial suspension diluted to 5 × 10^4^ conidia/ml in 0.2% gelatin was sprayed rice seedlings on 3–4 leaf stage. Diseased leaves were imaged 5 days after spray inoculation [54]. All experiments above were independently repeated three times, with three replicates each time. All statistical analyzes were performed with Duncan’s test (P < 0.01).

### Yeast two-hybrid assay

The coding sequence of each gene was amplified from full-length cDNA with the primers listed in Supporting Information Table S3. Amplified products were cloned into the bait vector pGBKT7 and prey vector pGADT7. After sequence verification, pairs of constructs were co-transformed into the yeast strain AH109 according to the instructions of the BD Matchmaker Library Construction & Screening Kits (Clontech, USA). Transformants grown on SD-Leu-Trp synthetic medium were transferred to SD-Leu-Trp-Ade-His synthetic medium. The yeast stains for positive and negative controls were from the Kit.

### Co-immunoprecipitation assays

The MoRac1- GFP construct was generated and transformed into wild-type 70–15 and Δ*Mofap7* mutant complemented with MoFap7-3×FLAG. Total proteins were extracted by lysis buffer (10 mM Tris-HCl [pH 7.5], 150 mM NaCl, 0.5 mM EDTA, 0.5% Triton X-100) and incubated with anti-GFP agarose (ChromoTek). Proteins bound to agarose were eluted after three times low salt and one time high salt buffer. Total proteins and elution proteins were detected with anti-FLAG (HuaAn) and anti-GFP (Abcam) antibodies using the ECL Supersignal system (Pierce, Rockford, IL) by western blot.

### In vitro GST pull-down assays

The full-length cDNA of *MoFAP7* was amplified and inserted into the vector pGEX4T-2 (GE Healthcare Life Science) to obtain the GST-MoFap7 plasmid. The full-length cDNAs of *MoMAT50, MoRAC1* and *MoCDC42* were amplified and inserted into the vector pET-32a to obtain the plasmids His-MoMst50, His-MoRac1 and His-MoCdc42, respectively. *E. coli* strain BL21 (DE3) cells expressing these plasmids were collected and treated with lysis buffer (10 mM Tris-HCl [pH 7.5], 150 mM NaCl, 0.5 mM EDTA, 0.5% Triton X-100). The bacterial lysate was subjected to SDS-PAGE and then to Coomassie blue staining to first the ensure expression of the GST or His fusion protein. After confirming expression, the soluble protein GST or GST-MoFap7 was incubated with 50 μl of glutathione agarose beads (Invitrogen) for 2 hours at 4°C. After the beads were washed five times, they were incubated with an equal amount of bacterial lysate containing His-MoMst50, His-MoRac1 or His-MoCdc42 for an additional hour at 4°C and eluted by boiling. Proteins in the elution were detected by immunoblotting using anti-His and anti-GST antibodies (HuaAn, Hangzhou, China).

### Fluorescence observation

The MoFap7 genomic DNA fragment without a terminator codon (TAA) was fused with eGFP cloned into the BamHI/SmaI site of the pKD9 [53] vector to generate pKD9-MoFap7-eGFP, and this plasmid was transformed into the Δ*Mofap7* mutant. To determine the localization, a nuclear localization signal called NLS-mCherry was transformed into the mutant. Green and red fluorescence was verified by confocal laser scanning microscopy with a Zeiss LSM780 during appressorial and mycelial development. Appressoria were incubated on hydrophobic plastic slides in humidified boxes at 25°C and mycelia were generated by incubating conidia in liquid CM for 24 h.

### CFW and DAB staining

To examine conidia morphology, conidia (~1 × 10^5^/ml) collected from CM agar plates and observed under an Eclipse 80i microscope (Nikon). The cell wall and conidia septum were visualized by CFW [Sigma-Aldrich) staining, as described by 57. For DAB staining assay, barley leaves infected with the strains at 32 hpi were stained with a 1 mg/ml DAB (Sigma-Aldrich] solution for 10 hours. The ethanol/acetic acid solution (ethanol/acetic acid = 98:2, v/v) was decolorized for 1 hour and observed under an Eclipse 80i microscope (Nikon).

### Mass spectrometry

The band corresponding to MoFap7-IP proteins was cut from SDS-PAGE gels and incubated in with 30% ACN/100 mM NH_4_HCO_3_ until the gels were destained. The gels were dried in a vacuum centrifuge. The in-gel proteins were reduced with dithiothreitol (10 mM DTT/100 mM NH_4_HCO_3_) for 30 min at 56°C, and were then alkylated with iodoacetamide (200 mM IAA/100 mM NH_4_HCO_3_) in the dark at room temperature for 30 minutes. Gel pieces were briefly rinsed with 100 mM NH_4_HCO_3_ and ACN. Gel pieces were digested overnight with 12.5 ng/μl trypsin in 25 mM NH_4_HCO_3_. Peptides were extracted three times with 60% ACN/0.1% TFA. The extracts were pooled and dried completely in a vacuum centrifuge.

Protein spots were incubated for 20 min in 30 mM potassium ferricyanide/100 mM sodium thiosulfate (1:1 v/v) and washed with Milli-Q spots until the gels were destained. The spots were incubated in 0.2 M NH_4_HCO_3_ for 20 min and then lyophilized. Each spot was digested overnight with 12.5 ng/µl trypsin in 25 mM NH_4_HCO_3_. Peptides were extracted three times with 60% acetonitrile (ACN)/0.1% trifluoroacetic acid(TFA). The extracts were pooled and dried completely in a vacuum centrifuge. A 50 μg sample was added to UA buffer, and DTT and iodoacetamide was added to 30 μl of SDT buffer (4% SDS, 100 mM DTT, 150 mM Tris-HCl [pH 8.0]). The detergent, DTT and other low-molecular-weight components were removed using UA buffer (8 M urea, 150 mM Tris-HCl [pH 8.0]) by repeated ultrafiltration (Microcon units, 10 kD). Then 100 μl iodoacetamide (100 mM IAA in UA buffer) was added to block reduced cysteine residues and the samples were incubated for 30 min in the dark. The filters were washed first with 100 μl UA buffer three times and then with 100 μl 25 mM NH_4_HCO_3_ buffer twice. Finally, the protein suspensions were digested with 4 μg of trypsin (Promega) in 40 μl of 25 mM NH_4_HCO_3_ buffer overnight at 37°C, and the resulting peptides were collected as a filtrate. LC-MS/MS analysis was performed on a Q Exactive mass spectrometer (Thermo Scientific) coupled to an Easy nLC (Proxeon Biosystems, now Thermo Fisher Scientific) for 60/120/240 min (determined by the project proposal).

MS/MS spectra were searched using the MASCOT engine (Matrix Science, London, UK; version 2.2) against a nonredundant International Protein Index Aarabidopsis sequence database (v3.85) (released at September 2011; 39,679 sequences) from the European Bioinformatics Institute (http://www.ebi.ac.uk/). For protein identification, the following options were used. Peptide mass tolerance = 20 ppm, MS/MS tolerance = 0.1 Da, Enzyme = Trypsin, Missed cleavage = 2, Fixed modification Carbamidomethyl (C), Variable modification Oxidation(M)
