# Supplementary material for: MoFap7, a ribosome assembly factor, is required for fungal development and plant colonization of Magnaporthe oryzae
Source: Virulence. 2019 Dec 9;10(1):1047–63. doi: 10.1080/21505594.2019.1697123 (PMC6930019; doi:10.1080/21505594.2019.1697123)
Supplement: Supplemental Material [file kvir-10-01-1697123-s001.zip › suppl files caption.docx]

**Fig. S1. MoFap7 or MoRps14 could not interact with itself.**

A and B Yeast two-hybrid analysis. The pair of plasmids pGBKT7–53 and pGADT7-T was used as the positive control. Plates were incubated at 30 °C for 4 days before being photographed.

**Fig. S2. Construction of pKO1B gene-deletion cassettes.**

The 5’ and 3’ flanking fragments of the targeted genes were separately amplified from genomic DNA with primers 5f/5r and 3f/3r. Primers 5r and 3f have 5’ tails homologous to the BAR cassette, whereas those for 5f and 3r are homologous to the vector. The amplified three fragments and the HindIII/XbaI-linearization vector pKO1B were fused together with the fusion enzyme. Homologous recombination produced a circular knockout vector, which is subsequently transformed into *A. tumefaciens*.

**Fig. S3. Site mutation verification**

A-J. Only the DNA sequence of E is from 3’-5’, and other DNA sequences are from 5’-3’

K. Genomic agarose gel electrophoresis results.

**Fig. S4.** **Growth of MoFap7 site mutant strains**

A.70-15 and the MoFap7 site mutation strains were grown on CM medium for 8 days (1#, 2#, 3# represents different transformants with the same site-directed mutagenesis).

B. Statistical analysis of the diameters of hyphae from the wild-type 70-15, the MoFap7 point mutant strains on CM. Asterisks denote statistical significances (P < 0.01).

**Fig. S5. Pull down results with Ladder**

A and C. The results of Pull down with Ladder.

B. The recombinant GST-MoFap7 or GST bound to glutathione Sepharose beads was incubated with E. coli cell lysate containing His-MoRac1. Eluted proteins were analyzed by immunoblot (IB) with the monoclonal anti-His and anti-GST antibodies.

**Fig. S6. Yeast two-hybrid assay**

Yeast two-hybrid assay for examining the interaction between MoFap7 MA andMoMst50. The transformants expressing AD-MoMst50 and empty BD, empty AD and BD- MoFap7^MA^, and empty AD and BD were used as negative control.

**Fig. S7. Identification of knockout mutants**

A. The targeted gene in the transformant was screened by PCR using the tubulin gene as a positive control. A characteristic band was amplified from the wild type strain and the ectopic transformant, indicating that the target gene was contained, whereas the band was not found in the null mutant. A unique recombinant DNA fragment labeled as a knockout event in the transformant was screened by PCR. A 1.5-2.5 kb band was amplified from the null mutant, whereas the wild-type strain and the ectopic transformant did not have the band.

B. MoFap7 locus and gene deletion vector.

C. Mutant and wild type were verified by Southern blot analysis. Genomic DNA was digested with PstI and separated with a 0.7% agarose gel. The DNA was hybridized with a 1.0 kb fragment(probe indicated in Fig. S7B) amplified from genomic DNA of *M. oryzae* wild-type strain 70-15.

**Fig. S8.** **Verification of the insertion sites of the site mutation strains.**

A-H Sequence alignments of the rice blast fungus genome database (http://fungi.ensembl.org/Magnaporthe_oryzae/Info/Index?db=core) after sequencing of the hiTAIL PCR product.

I. qPCR to verify the copy number of the site mutation strains. β-tubulin was used as control.

**Table.S1** Fap7 interacting proteins as identified by co-immunoprecipitation experiments.

**Table.S2** Fap7 interacting protein identified by yeast two-hybrid screen library.

**Table.S3** List of primers used in this study.

**Table.S4** Procedure of the high-efficiency thermal asymmetric interlaced PCR (hiTAIL-PCR).
