# Supplementary material for: MoFap7, a ribosome assembly factor, is required for fungal development and plant colonization of Magnaporthe oryzae
Source: Virulence. 2019 Dec 9;10(1):1047–63. doi: 10.1080/21505594.2019.1697123 (PMC6930019; doi:10.1080/21505594.2019.1697123)
Supplement: Supplemental Material [file kvir-10-01-1697123-s001.zip › Table S1.docx]

Table S1: Fap7 interacting proteins as identified by co-immunoprecipitation
experiments.

|  | **Score** | **Expect Value** |
| --- | --- | --- |
| Rps14 | 57.61 | 7.43E-05 |
| Rho1 | 32.49 | 1.10E-01 |
| Sep3 | 43.69 | 3.19E-03 |
| Sep6 | 27.06 | 5.27E-01 |
| Cdc15 | 20.06 | 1.35E+00 |
| Pmk1 | 25.59 | 1.98E-01 |
