# Supplementary material for: MoFap7, a ribosome assembly factor, is required for fungal development and plant colonization of Magnaporthe oryzae
Source: Virulence. 2019 Dec 9;10(1):1047–63. doi: 10.1080/21505594.2019.1697123 (PMC6930019; doi:10.1080/21505594.2019.1697123)
Supplement: Supplemental Material [file kvir-10-01-1697123-s001.zip › Table S2.docx]

| ***S.cerevisiae*** | ***M.oryzae*** | **reference** |
| --- | --- | --- |
| Ypt7 | MGG_08144 | Liu et al., 2015 |
| Mst50 | MGG_05199 | Park et al., 2006 |
| Cdc42 | MGG_00466 | Zheng et al., 2009 |
| Rac1 | MGG_02731 | Chen et al., 2008 |
| Rps14 | MGG_05238 | Antunez and Woolford, 2003 |
| Rho1 | MGG_07176 | Drgonova et al., 1999 |

**Table S2: Fap7 interacting protein identified by yeast two-hybrid screen library**
