# Supplementary material for: MoFap7, a ribosome assembly factor, is required for fungal development and plant colonization of Magnaporthe oryzae
Source: Virulence. 2019 Dec 9;10(1):1047–63. doi: 10.1080/21505594.2019.1697123 (PMC6930019; doi:10.1080/21505594.2019.1697123)
Supplement: Supplemental Material [file kvir-10-01-1697123-s001.zip › Table S3 Primers in this study.docx]

**Table S3 Primers in this study**

| Name Sequence (5’ - 3’) | |
| --- | --- |
| Primers used for gene knockout | |
| BAR-F | AGAAGATGATATTGAAGGAGC |
| BAR-R | CTAAATCTCGGTGACGGGCAG |
| FAP7up-F | CCGGGGATCCTCTAGA ACTGCCCTGGAGACTTATACC |
| FAP7up-R | CTCCTTCAATATCATCTTCT CCTAATCGTTGCGTAAACACTG |
| FAP7dn-F | TGCCCGTCACCGAGATTTAG GCCTCTCCTTTTCTCGTGATAC |
| FAP7dn-R | GGCCAGTGCCAAGCTT GTGCCCGTCTGTCAAATACC |
| FAP7inner-F | TGTCTATGTCCCCGAAAAGG |
| FAP7inner-R | CATGATCTCCGAGTCCAGGT |
| FAP7upyz-F | AGCATGGACAACCTGCTCAG |
| FAP7upyz-R | AGGGCGAACTTAAGAAGGTATG |
| Primers used for yeast two-hybrid assay | |
| FAP7-AD-F | GGAGGCCAGTGAATTCATGGCGACAAGATCTCTGCCAA |
| FAP7-AD-R | CGAGCTCGATGGATCCGACGCCATTGTCCTTCTTCCAC |
| FAP7-BD-F | CATGGAGGCCGAATTCATGGCGACAAGATCTCTGCCAA |
| FAP7-BD-R | GCAGGTCGACGGATCCGACGCCATTGTCCTTCTTCCAC |
| RHO1-AD-F | GGAGGCCAGTGAATTCATGGCCGAAATCCGCCGCAAG |
| RHO1-AD-R | CGAGCTCGATGGATCCGAGGATGAGGCACTTCTTCTTC |
| RPS14-AD-F | GGAGGCCAGTGAATTCATGCCTCCCAAGAAGACTCAAC |
| RPS14-AD-R | CGAGCTCGATGGATCCGAGACGACGACCACGGCGACCA |
| RAC1-AD-F | GGAGGCCAGTGAATTCATGGCCGCCCCTGGGGTTCAGT |
| RAC1-AD-R | CGAGCTCGATGGATCCCAGAATGGTGCACTTTGAC |
| CDC42-AD-F | GGAGGCCAGTGAATTCATGGTGGTTGCAACGATT |
| CDC42-AD-R | CGAGCTCGATGGATCCAAGGATCAGGCACTTTTTGGAT |
| STE50-AD-F | GGAGGCCAGTGAATTCATGAGCTTCAACACGGGGACGG |
| STE50-AD-R | CGAGCTCGATGGATCCTATTATTCCTCCTGGGGGAT |
| Primers used in fluorescent observation | |
| FAP7-GFP-F | CACAATGGCCGGATCCATGGCGACAAGATCTCTGCC |
| FAP7-GFP-R | TGCTCACCATCCCGGGGACGCCATTGTCCTTCTTCC |
| Primers used for pull-down | |
| CDC42-His-F | CAAGGTCGACAAGCTTATGGTGGTTGCAACGATT |
| CDC42-His-R | GTGCGGCCGCAAGCTTAAGGATCAGGCACTTTTTGGAT |
| RAC1-His-F | CAAGGTCGACAAGCTTATGGCCGCCCCTGGGGTTCAGT |
| RAC1-His-R | GTGCGGCCGCAAGCTTCAGAATGGTGCACTTTGAC |
| MST50-His-F | CAAGGTCGACAAGCTTATGAGCTTCAACACGGGGACGG |
| MST50-His-R | GTGCGGCCGCAAGCTTTATTATTCCTCCTGGGGGAT |
| FAP7-GST-F | GGTTCCGCGTGGATCCGGTGGTGGTGGTGGTATGGCGACAAGATCTCTGCC |
| FAP7-GST-R | GGAATTCCGGGGATCCGACGCCATTGTCCTTCTTCC |
| Primers used for Co-IP | |
| Rac1-GFP-F | ATCACAATGGCCGGATCCATGGCCGCCCCTGGGGTTCAGT |
| Rac1-GFP-R | CATCCCGGGGATGGATCCCAGAATGGTGCACTTTGAC |
| Fap7-Flag-F | CACAATGGCCGGATCCATGGCGACAAGATCTCTGCC |
| Fap7-Flag-R | CCCCGGGGATGGATCCGACGCCATTGTCCTTCTTCC |
| Primer used for hiTAIL-PCR | |
| LAD1 | ACGATGGACTCCAGAGCGGCCGCVNVNNNGGAA |
| LAD2 | ACGATGGACTCCAGAGCGGCCGCVNVNNNGGAA |
| LAD3 | ACGATGGACTCCAGAGCGGCCGCHNVNNNCCAC |
| LAD4 | ACGATGGACTCCAGAGCGGCCGCVVNVNNNCCAA |
| LAD5 | ACGATGGACTCCAGAGCGGCCGCBDNBNNNCGGT |
| AC1 | ACGATGGACTCCAGAG |
| R1 | GGCACTGGCCGTCGTTTTACAAC |
| R2 | AACGTCGTGACTGGGAAAACCCT |
| R3 | CCCTTCCCAACAGTTGCGCA |
| Primer used for qPCR | |
| qRT-tubulin-F | ACAACTTCGTCTTCGGTCAG |
| qRT-tubulin-R | GTGATCTGGAAACCCTGGAG |
| qRT-Fap7-F | CTCTTGGATGCGATCGAGGATG |
| qRT-Fap7-R | AAGCATGCCAGTCGATGATGC |
