# Supplementary material for: MoFap7, a ribosome assembly factor, is required for fungal development and plant colonization of Magnaporthe oryzae
Source: Virulence. 2019 Dec 9;10(1):1047–63. doi: 10.1080/21505594.2019.1697123 (PMC6930019; doi:10.1080/21505594.2019.1697123)
Supplement: Supplemental Material [file kvir-10-01-1697123-s001.zip › Table S4 HiTAIL-PCR.docx]

**Table S4 Procedure of the high-efficiency thermal asymmetric interlaced PCR(hiTAIL-PCR).**

1. **hiTAIL-PCR reaction system**

| Reagent | Amount in ready  Reaction (µl) | Amount in primary  reaction (µl) | Amount in secondary  reaction (µl) |
| --- | --- | --- | --- |
| ddH_2_O | 14.4 | 17.9 | 17.9 |
| 10 × PCR buffer | 2 | 2.5 | 2.5 |
| dNTPS (10 mM each) | 0.4 | 0.5 | 0.5 |
| MgCl_2_ (25 mM) | 1.2 | 1.5 | 1.5 |
| SP (20 µM) | 0.3 | 0.3 | 0.3 |
| LAD (20 µM) | 1 | － | － |
| AC1 (20 µM) | － | 1 | 1 |
| Taq (5 U/µL) | 0.2 | 0.25 | 0.25 |
| DNA Template | 0.5 (30 nmol genome DNA) | 1 (40 ×ready reaction  product diluent) | 1 (10 ×primary reaction product diluent) |

Ex Taq DNA polymerase kit with 10× PCR buffer containing 20 mM MgCl_2_ (Takara-Bio, Dalian,

China) was used for the PCR.

**2. Thermal conditions for hiTAIL-PCR**

| Reaction | File No. | Thermal cycling condition | Cycle No. |
| --- | --- | --- | --- |
| Pre-amplification | 1 | 93ºC 2 min 95ºC1 min | 1 |
|  | 2 | 94ºC30 s 60ºC1 min 72ºC3 min | 10 |
|  | 3 | 94ºC30 s 25ºC2 min ramping to 72ºC over 2 min 72ºC 3 min | 1 |
|  | 4 | 94ºC20 s 60ºC1 min 72ºC3 min | 25 |
|  | 5 | 72ºC5 min | 1 |
| primary | 1 | 94ºC2 min | 1 |
|  | 2 | 94ºC20 s 66ºC1 min 72ºC3 min 94ºC20 s  66ºC1 min 72ºC3 min 94ºC20 s 50ºC1 min 72ºC3 min | 12 |
|  | 3 | 72ºC5 min | 1 |
| Secondary | 1 | 94ºC20 s 66ºC1 min 72ºC3 min 94ºC20 s  66ºC1 min 72ºC3 min 94ºC20 s 50ºC1 min 72ºC3 min | 8 |
|  | 2 | 72ºC5 min | 1 |
