# Supplementary figures and images for: MoFap7, a ribosome assembly factor, is required for fungal development and plant colonization of Magnaporthe oryzae
Source: Virulence. 2019 Dec 9;10(1):1047–63. doi: 10.1080/21505594.2019.1697123 (PMC6930019; doi:10.1080/21505594.2019.1697123)

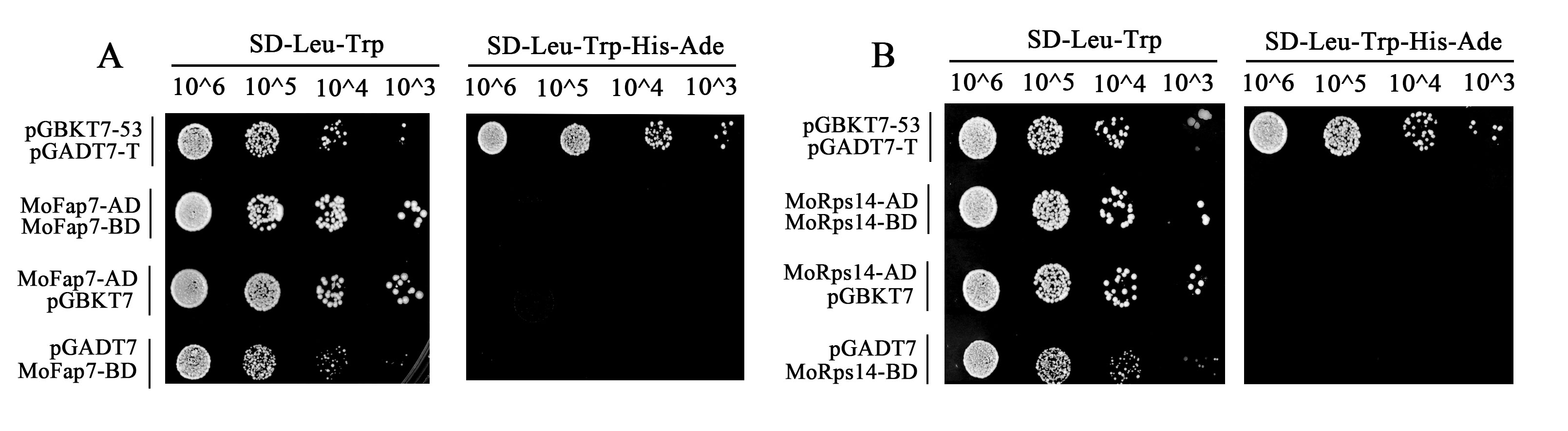

Supplement: Supplemental Material [file kvir-10-01-1697123-s001.zip › Fig-S1.jpg]

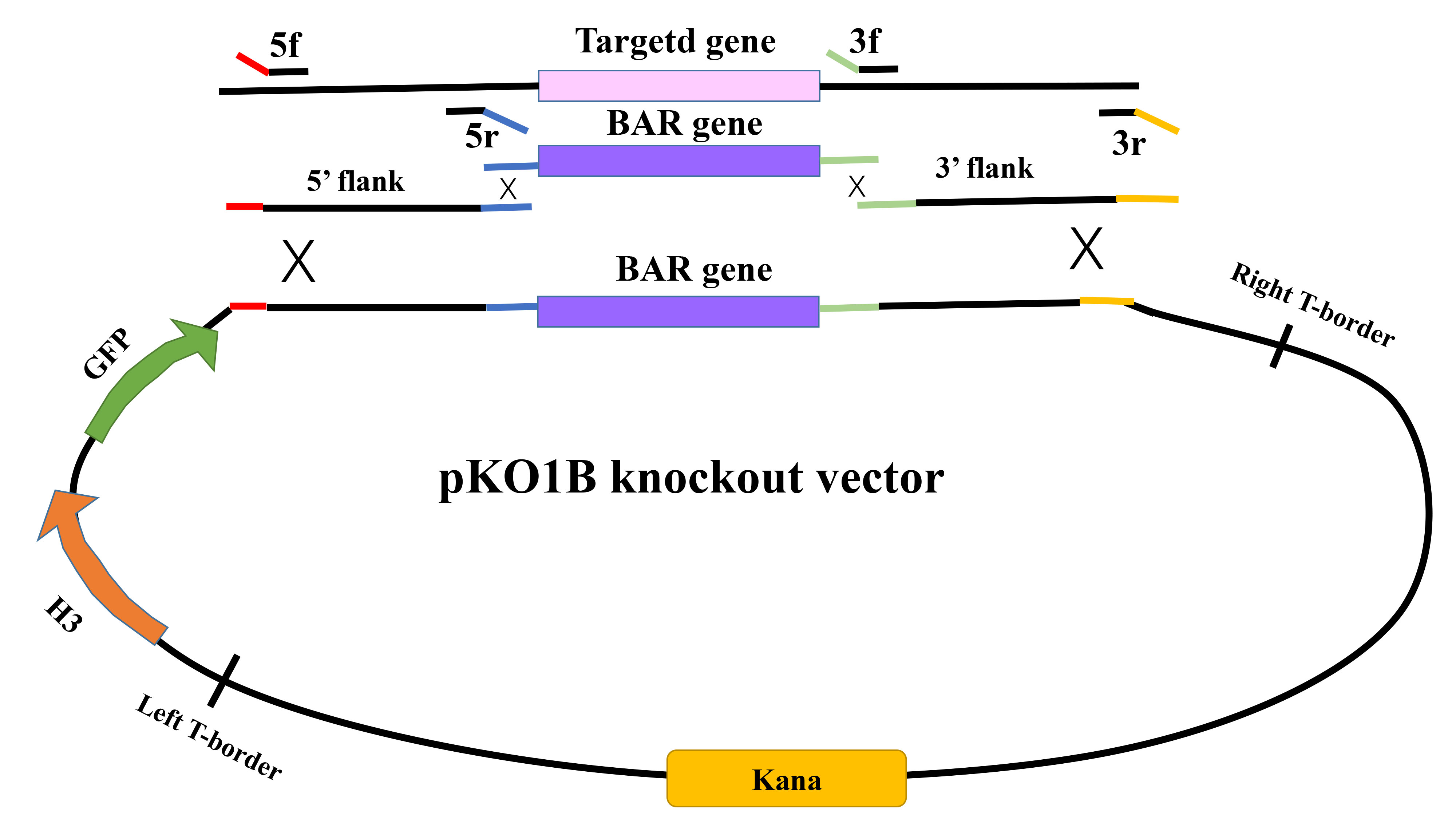

Supplement: Supplemental Material [file kvir-10-01-1697123-s001.zip › Fig-S2.jpg]

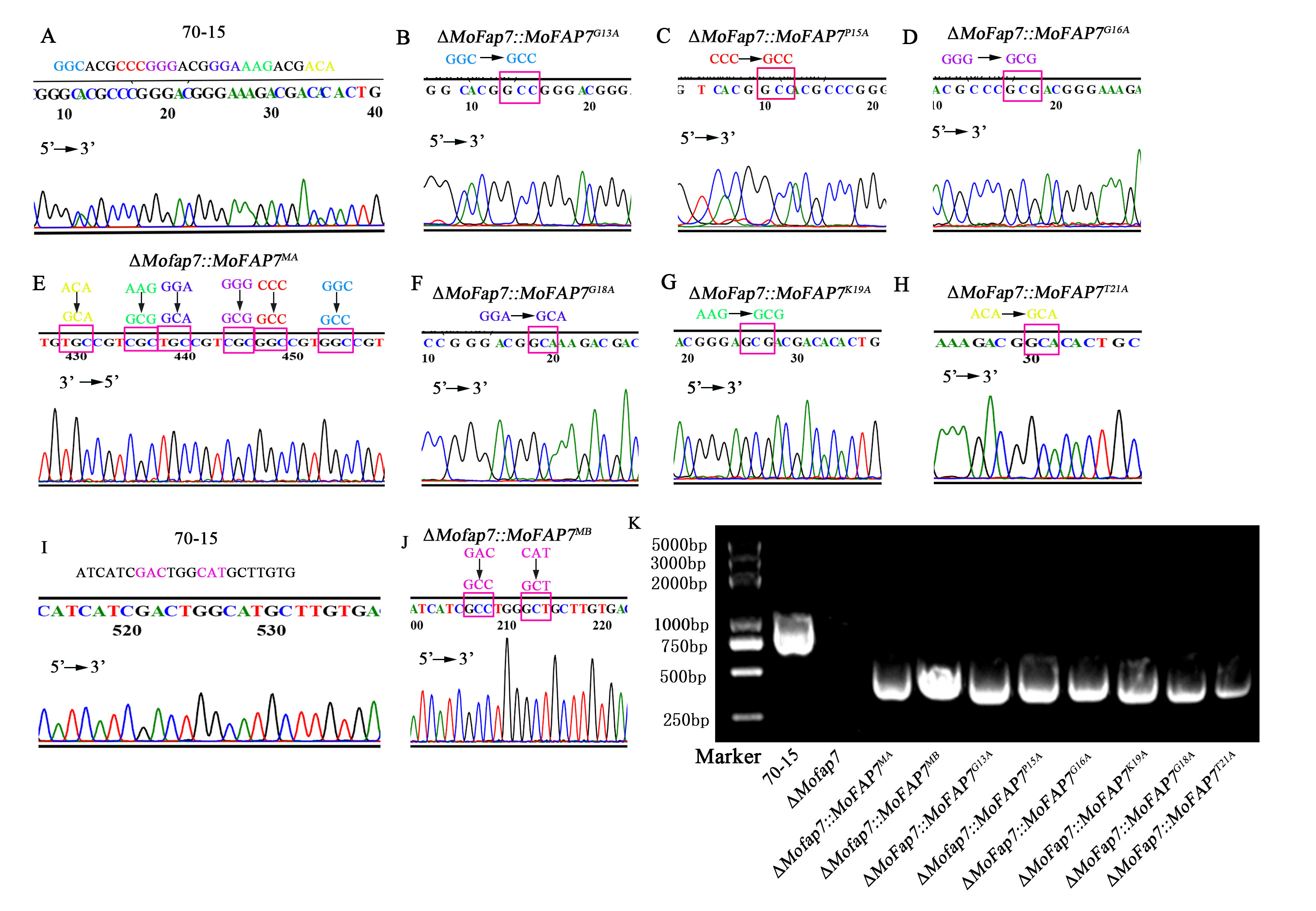

Supplement: Supplemental Material [file kvir-10-01-1697123-s001.zip › Fig-S3.jpg]

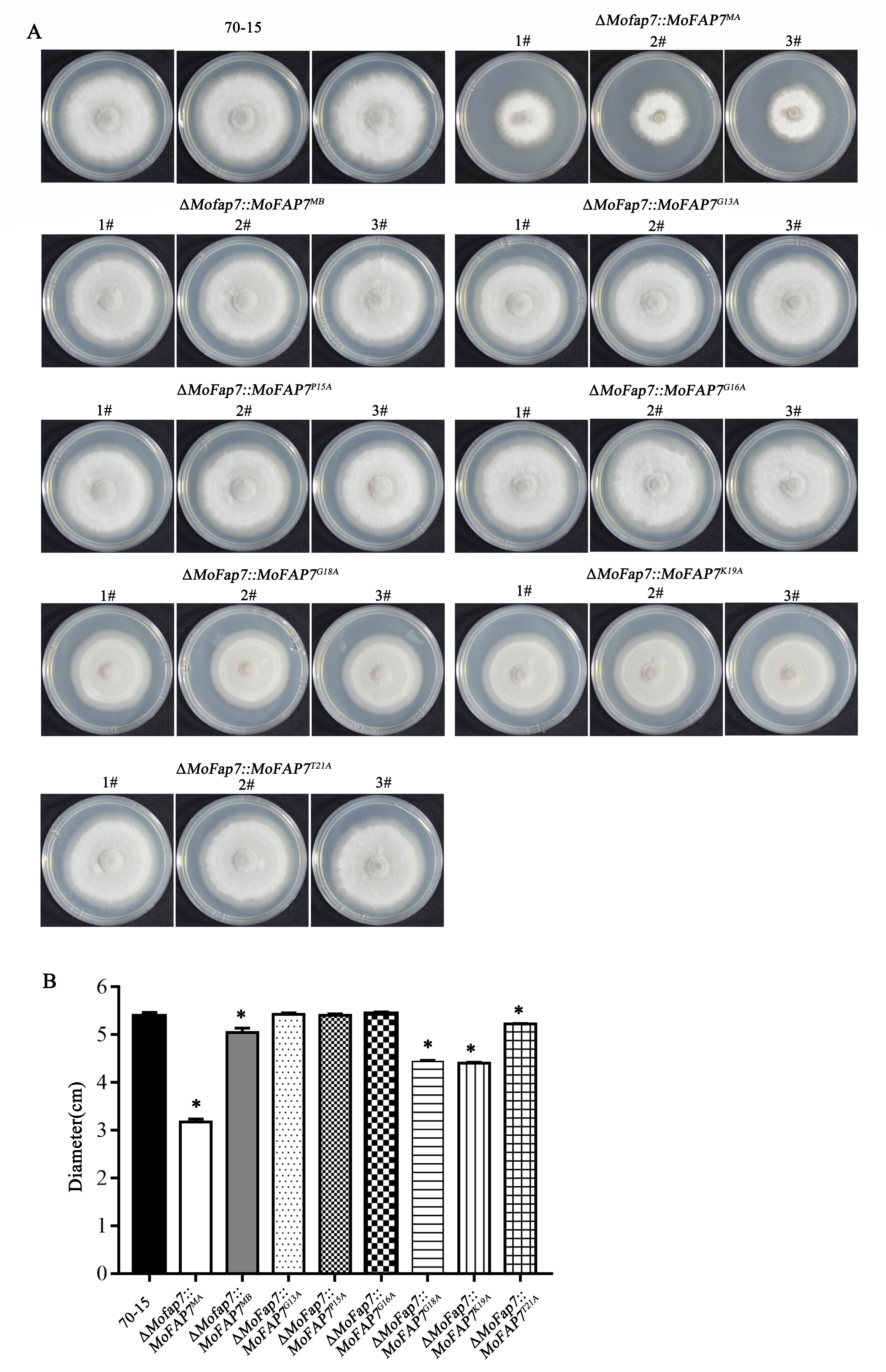

Supplement: Supplemental Material [file kvir-10-01-1697123-s001.zip › Fig-S4.jpg]

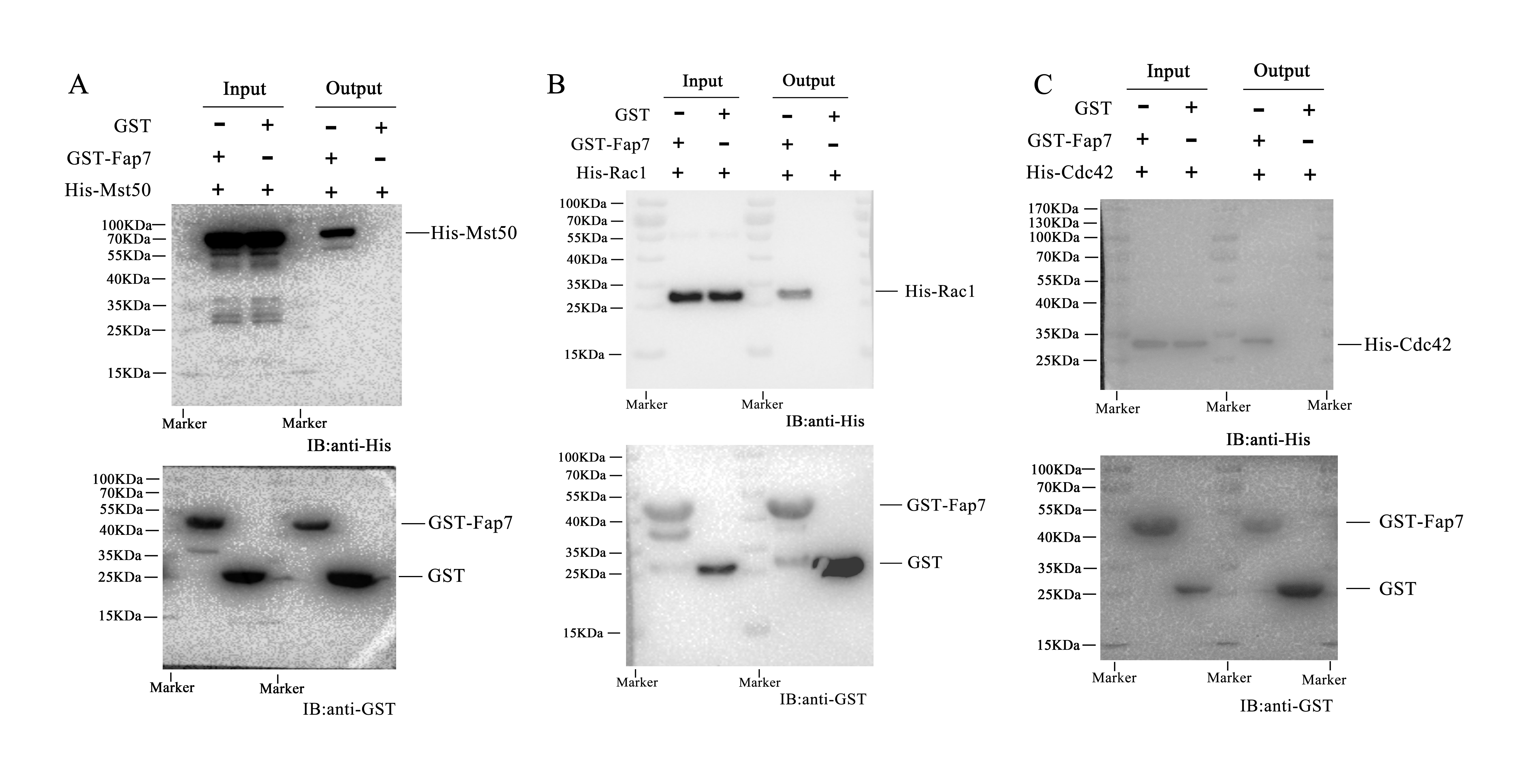

Supplement: Supplemental Material [file kvir-10-01-1697123-s001.zip › Fig-S5.jpg]

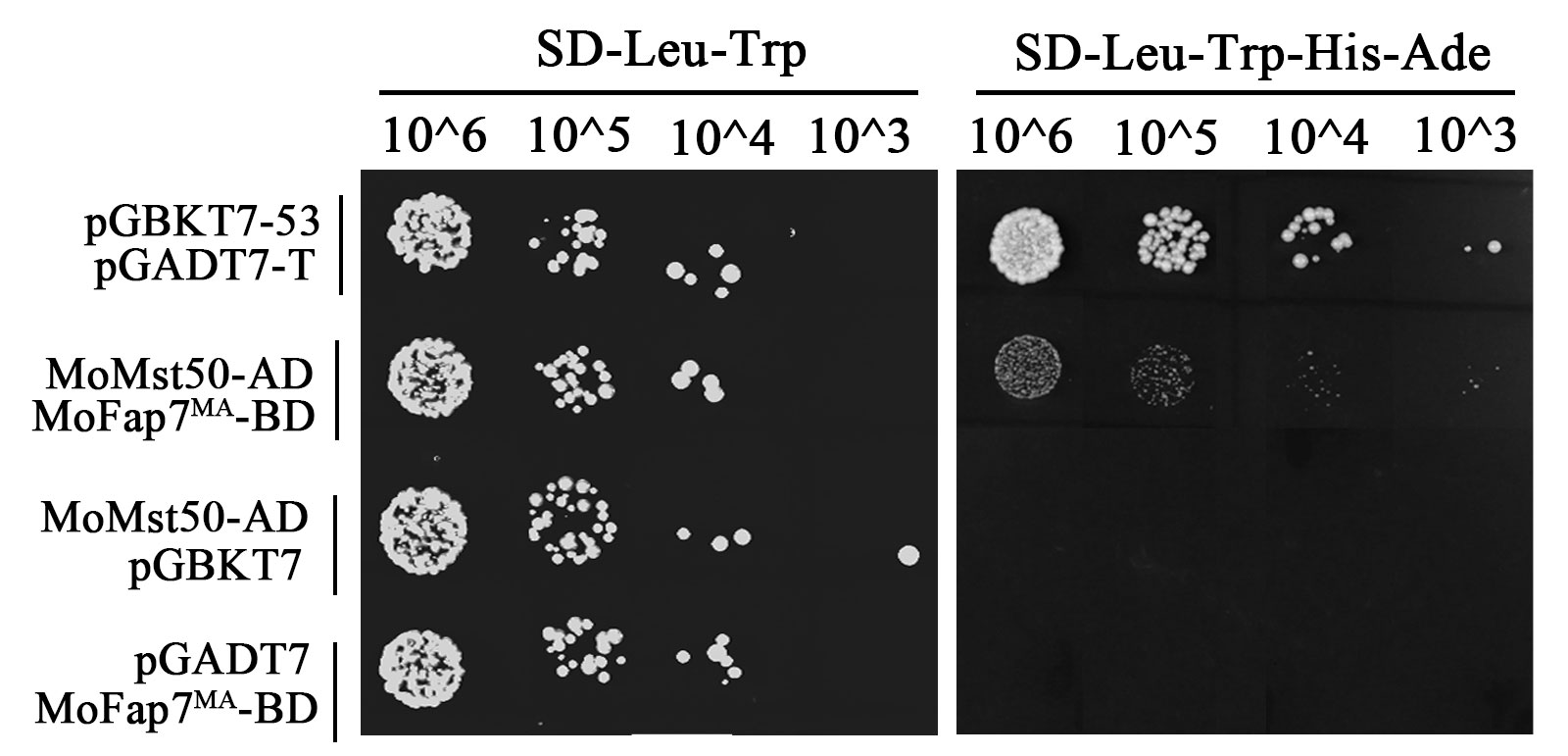

Supplement: Supplemental Material [file kvir-10-01-1697123-s001.zip › Fig-S6.jpg]

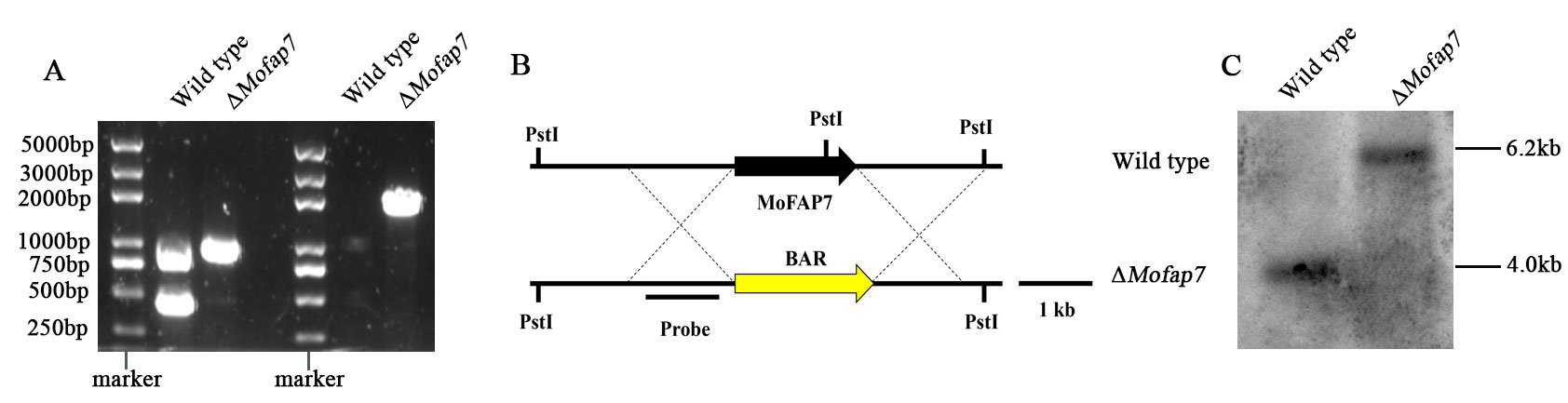

Supplement: Supplemental Material [file kvir-10-01-1697123-s001.zip › Fig-S7.jpg]

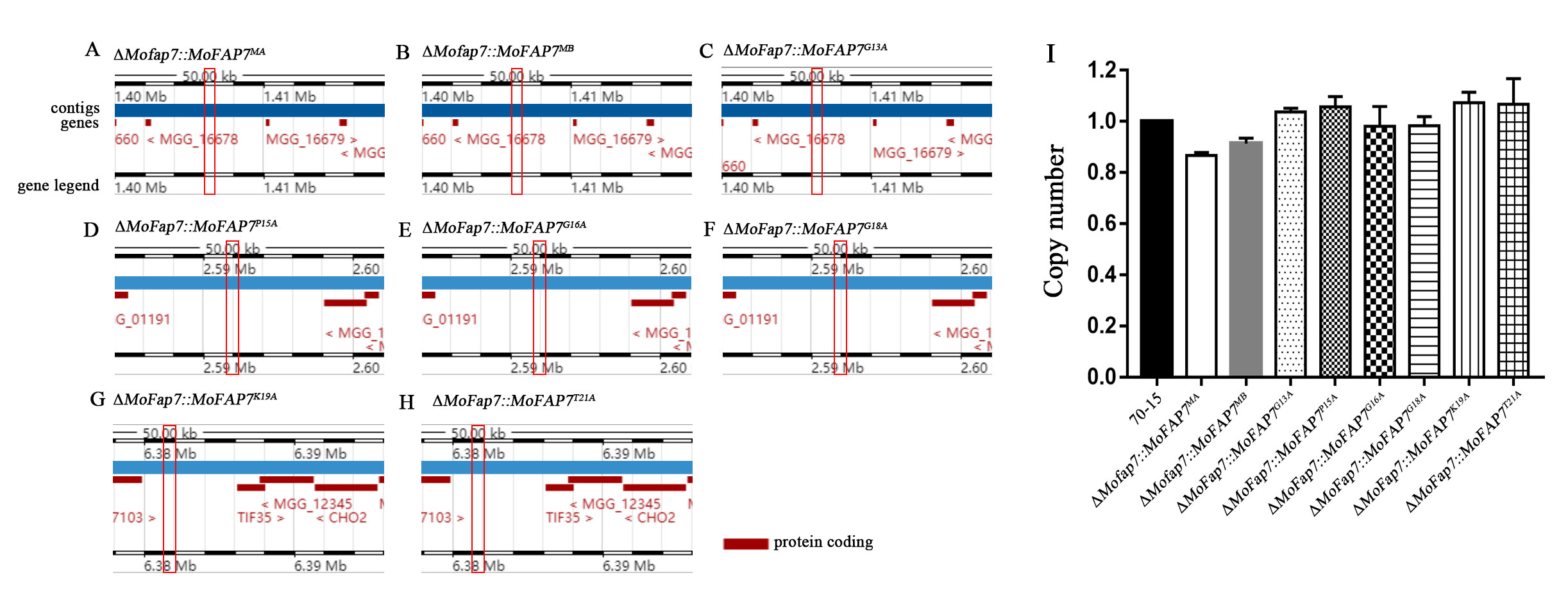

Supplement: Supplemental Material [file kvir-10-01-1697123-s001.zip › Fig-S8.jpg]
